# Alzheimer's disease and mixed pathologies as a hidden contributor to fatal hypothermia: A large‐scale forensic autopsy‐based study

**DOI:** 10.1111/bpa.70051

**Published:** 2025-11-30

**Authors:** Shojiro Ichimata, Koji Yoshida, Ryo Tanaka

**Affiliations:** ^1^ Department of Legal Medicine, Faculty of Medicine University of Toyama Toyama Japan; ^2^ Department of Neurology Toyama University Hospital Toyama Japan

**Keywords:** Alzheimer's disease, hypothermia, neuropathology, older adults, wandering

## Abstract

There is a paucity of autopsy‐based studies providing detailed neuropathological characteristics of fatal hypothermia cases, particularly in trauma‐associated cases. Hence, this study investigated 2054 serial autopsy cases in which histopathological examination of all organs, including the brain, could be performed. We identified 168 cases (75 female and 93 male patients) of fatal hypothermia and examined the clinical and neuropathological characteristics in these cases. Patients aged ≥65 years constituted 80% of the cohort (135 cases). Cognitive impairment (CI) was identified in 39% of cases with available clinical histories, and approximately half of these cases were presumed to have developed hypothermia while wandering, based on clinical history and the circumstances of discovery. Alzheimer's disease was the most commonly identified pathology, affecting 40% of the total and approximately two‐thirds of patients aged ≥80 years. CI caused by multiple pathologies, especially two, was more frequently observed than by a single pathology. The most common manner of exposure to cold temperatures was accidents (120 cases), including 35 cases with multiple traumatic injuries, most likely resulting from falls. In these cases, the trauma was considered the primary cause of immobility, leading to subsequent exposure to cold environments. Importantly, 30 (86%) of these trauma‐associated cases showed one or more neuropathological conditions, and CI was documented in 13, with four presumed to have fallen while wandering. Notable neuropathological manifestations were also observed in eight of the 33 cases of patients aged less than 65 years. Our results demonstrate that neurodegenerative diseases, especially Alzheimer's disease, are underlying conditions in fatal hypothermia in the elderly and in relatively younger individuals, suggesting that they may contribute to its onset. These findings highlight the necessity for comprehensive neuropathological examination in all forensic autopsy cases of hypothermia.

## Graphical abstract

We investigated neuropathological manifestations of hypothermia in 168 cases. Patients aged 65 years or more constituted 80% of the total cohort. Alzheimer's disease was the most common comorbidity, affecting 40% of all patients.

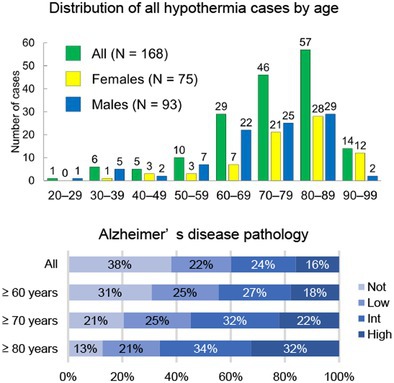

## 1 BACKGROUND

Hypothermia is a morbidity with a high mortality rate, in which the core body temperature falls below 35°C [1]. Several factors, including environmental conditions and individual characteristics, are central to the development of the condition. In general, older adults are considered to be at higher risk because of a combination of factors [1]. A large hospital‐based report demonstrated that accidental hypothermia was more likely to develop in individuals aged 65 years or more (80%) [2]. Furthermore, we recently reported on hypothermia cases in forensic settings, with 81% of them involving patients within this age group [3]. These results indicate that hypothermia is a condition that predominantly affects older adults. As population aging is considered to be a global issue, more knowledge on the clinicopathological features of hypothermia in older adults is required.

We previously reported that forensic autopsy cases of unnatural deaths often involve various neurodegenerative diseases (NDDs) [4, 5, 6, 7]. NDDs are closely associated with aging [8], and may be a risk factor for hypothermia in older adults. Consistent with this hypothesis, dementia with wandering has been shown to be a primary cause of hypothermia in forensic cases [9, 10, 11]. In addition, several neuropathological conditions, including cerebrovascular diseases, NDDs, and other rare conditions, can cause motor disturbances [12, 13, 14]. Consequently, these patients are considered prone to trauma caused by falls [15], which pose further risks for the development of hypothermia through blood loss and reduced ability to move away from low‐temperature environments [1]. Priemer et al. reported that in many forensic autopsy cases, several NDDs had been associated with falls [16], thus supporting this hypothesis. Additionally, the presence of α‐synucleinopathy may impair heat production, making it a potential risk factor for hypothermia [17]. Taken together, these findings highlight the need for a comprehensive analysis of data from a large number of autopsies of hypothermia cases, including trauma‐positive cases associated with falls, to reveal the underlying neuropathological conditions among fatal hypothermia cases. However, there is a paucity of autopsy‐based studies providing detailed neuropathological characteristics of hypothermia, and the NDDs associated with this condition are still poorly understood.

To elucidate this, we performed a comprehensive neuropathological study using modern immunohistochemistry laboratory methods in a series of fatal hypothermia cases. Our objective was to evaluate the type and frequency of neuropathologic conditions that were the cause of fatal hypothermia and their association with clinical symptoms and fall‐related trauma, as well as whether they can contribute to the development of hypothermia in adults younger than 65 years.

## 2 METHODS

### 2.1 Participants

We evaluated the archives of 2054 cases (802 female and 1252 male patients), who were autopsied in our department from January 2011 to March 2024, and in whom all the organs, including the brain, could be sampled and histopathologically analyzed. All deaths occurred in Toyama Prefecture and its areas, a heavy snowfall region in north‐central Japan. The patients' demographic and clinical data, including their cause of death, were extracted from the forensic medical records, police examinations, family members' contributions, or their primary physician if a patient's record indicated clinic visits. Based on these data, we retrieved the cases where hypothermia was presumably the leading cause of death. Fall‐related injuries were classified as positive if the following criteria were met: (1) the circumstances of discovery suggested that the patient had fallen; (2) the severity of the injury was sufficient to cause immobility; and (3) the injury itself was not considered the direct cause of death. Cases with a clinical diagnosis of cognitive impairment (CI), or with a strong suspicion of it based on information from family members or caregivers, were considered CI‐positive.

### 2.2 Tissue sampling and pathological assessments

All brains underwent fixation in 20% buffered formalin for 2–4 weeks before standard sampling was performed at our department, as outlined in a previous study [5]. All of the brains were sectioned before staining with Luxol fast blue, hematoxylin and eosin, and Gallyas–Braak. Routine immunohistochemical screening was performed on samples from the frontal lobe, hippocampus and temporal cortex (including the posterior hippocampus at the level of the lateral geniculate nucleus through the inferior temporal gyrus), basal ganglia, midbrain, and medulla oblongata in all cases. Immunohistochemistry was performed using the following primary antibodies: phosphorylated tau (clone AT8, 1:1000; Endogen, Woburn, MA, USA), phosphorylated α‐synuclein (clone LB508, 1:500; Zymed, San Francisco, CA, USA), TDP‐43 (1:5000; Proteintech Group, Chicago, IL, USA), and Aβ (Clone 6F/3D, 1:50, Dako, Glostrup, Denmark). Antibody binding was identified using a biotin‐streptavidin detection system (Nichirei) with 3,3′‐diaminobenzidine as the chromogenic substrate. Staining for three‐ (RD3, clone 8E6/C11, Merck Millipore, Billerica, MA, USA) and four‐repeat tau (RD4, clone 1E1/A6, Merck Millipore) was performed in AT8‐positive cases suspected of tauopathies other than AD. Furthermore, if the initial immunohistochemical examination indicated the presence of NDDs, additional staining necessary for the final diagnosis was performed according to the criteria below.

Pathological staging of neurofibrillary tangles (NFTs) was performed as previously described by Braak, which revealed the NFT burden with AT8 and Gallyas–Braak staining [18]. Neuritic plaque density was assessed according to the Consortium to Establish a Registry for Alzheimer's Disease (CERAD) criteria and by applying thioflavin‐S and Aβ immunostaining [19]. The extent of senile plaque development in a brain was estimated by applying the criteria detailed by Thal et al. [20]. Based on these outcomes, AD‐related neuropathological changes were categorized into four groups according to the National Institute on Aging–Alzheimer's Association (NIA‐AA) guidelines: Not, Low, Intermediate, and High [21, 22]. Lewy body disease (LBD) pathology was analyzed as per the Third Consensus Guidelines for Dementia with Lewy Body, and Braak staging, which reflects the progression of Parkinson's disease‐related pathology, was detected using α‐synuclein immunohistochemistry [23, 24, 25]. Argyrophilic grains were identified using Gallyas–Braak staining, and pathological argyrophilic grain disease (AGD) was staged with the system described by Saito et al. [26]. We applied the National Institute of Neurological Disorders and Stroke (NINDS) criteria and those of the Rainwater Charitable Foundation to confirm a neuropathological diagnosis of progressive supranuclear palsy (PSP) [27, 28]. A pathological type of TDP‐43 proteinopathy was staged as per the criteria of the limbic‐predominant age‐related TDP‐43 encephalopathy neuropathologic changes (LATE‐NC) pathology [29]. Vascular‐related CI was suspected when moderate or severe arteriolosclerosis and/or subcortical cerebral infarct(s) were found [30, 31]. Furthermore, a diagnosis of chronic Wernicke encephalopathy was confirmed based on the findings of neuronal loss and gliosis in the mamillary bodies [32]. In addition, pellagra encephalopathy was suspected when central chromatolysis was evident in pontine nucleus neurons [33]. These conditions were attributed to malnutrition‐related pathologies. Moreover, in patients with a history of chronic alcoholism, a diagnosis of cerebellar degeneration was made when cerebellar atrophy with a substantial decrease in the number of Purkinje cells and an increase in that of Bergmann glia was detected [34, 35].

### 2.3 Blood and serum sample analysis

During the autopsies, drug intake was screened with Triage DOA kits (Sysmex, Kobe, Japan) or SIGNIFYER kits (Sysmex), and a gas chromatography‐based autoanalyzer (GC‐2014, Shimadzu, Kyoto, Japan) was applied to determine the blood ethanol concentrations at a police laboratory in cases of suspected drug abuse.

### 2.4 Statistical analysis

IBM SPSS Statistics version 29 (SPSS Inc., Chicago, IL) was used for data analysis. A two‐tailed *p*‐value <0.05 was regarded as statistically significant. We applied Fisher's exact test for the comparative analysis of categorical variables (sex, symptom presence, and clinicopathological findings) and the Mann–Whitney *U*‐test for continuous variables (age at death and body mass index).

## 3 RESULTS

### 3.1 Clinical and demographic data

**FIGURE 1:**
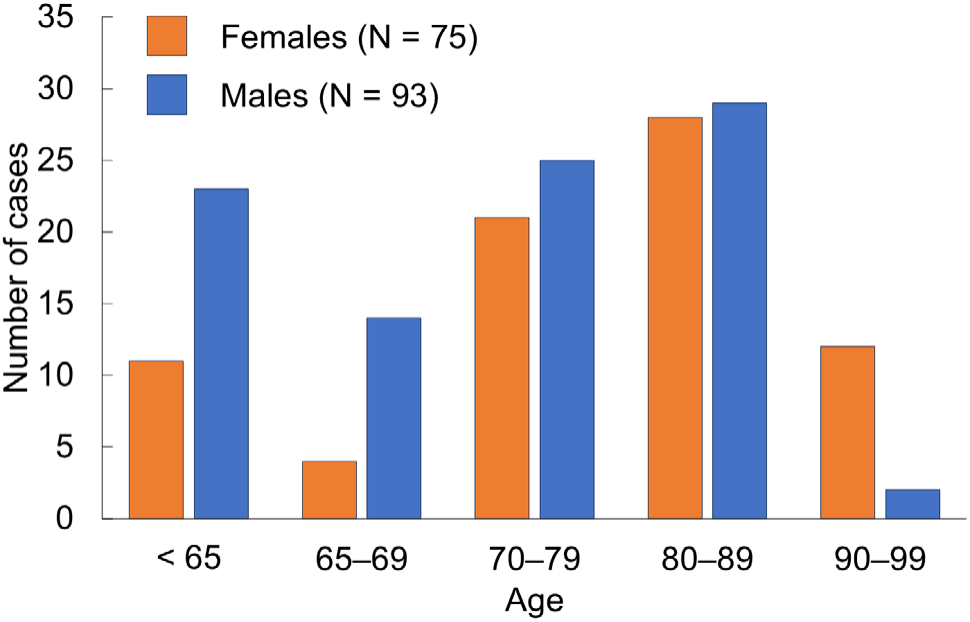
Distribution of cases by age.

We identified 168 cases of fatal hypothermia (8.2% of the total cases; 9.4% female and 7.4% male patients). Table 1 provides a summary of the clinicopathologic features of patients in the cases. Table S1 details all clinicopathological findings, and the age distribution is shown in Figure S1. Further, the distribution of cases by age is shown in Figure 1.

**TABLE 1:** Summary of all hypothermia cases in this study.

	All (*N* = 168)	Female (*N* = 75)	Male (*N* = 93)	*p‐*Value^a^
Number of cases	168	75	93	
Mean age (range)	74.0 ± 14.3 (24–99)	77.9 ± 12.9 (32–99)	70.9 ± 14.6 (24–92)	**<0.01**
Mean BMI (range)	19.4 ± 3.3 (10.3–28.2)	18.8 ± 3.3 (11.9–28.2)	19.8 ± 3.2 (10.3–27.1)	**0.04**
Brain weight (g) (range)	1319.2 ± 143.7 (1032–1780)	1226.8 ± 106.7 (1032–1586)	1393.7 ± 125.2 (1151–1780)	
Outdoor/indoor (%)	121/47 (72/28)	54/21 (72/28)	67/26 (72/28)	1
Manner of exposure to low temperature conditions (%)				
Illness	21 (13)	9 (12)	12 (13)	1
Accident	120 (71)	47 (63)	73 (78)	**0.03**
Suicide	24 (14)	17 (23)	7 (8)	**<0.01**
Unknown	3 (2)	2 (3)	1 (1)	0.59
Complications (%)^b^				
Cognitive impairment	60 (36)	31 (46)	29 (34)	0.18
Wandering	31 (20)	14 (21)	17 (20)	1
Psychiatric visits	14 (9)	11 (16)	3 (4)	**<0.01**
Chronic alcoholism	6 (4)	0	6 (6)	**0.03**
Toxicology testing (%)				
Blood ethanol‐positive	17 (10)	1 (1)	16 (17)	**<0.01**
Drug‐positive^c^	22 (13)	11 (15)	11 (12)	0.65

*Note*: Boldface signifies the values that are significant at *p* < 0.05.

Abbreviation: BMI, body mass index.

^a^ Female versus male patients, evaluated using Fisher's exact test or Mann–Whitney *U* test.

^b^ Frequency in 153 cases (68 female and 85 male patients), in whom the clinical information was available.

^c^ Summary of the results of screening tests using urine and detailed examinations using blood.

The mean age of female patients was significantly higher than that of males. The percentage of patients aged 65 years or more composed 80% (135 cases) (Figure 1), with a tendency to be higher in females (87%, 65 cases) than in male patients (75%, 70 cases) (*p* = 0.08). Furthermore, the mean body mass index was significantly lower in the former. Fatal hypothermia occurred more frequently in outdoor settings. The manner of exposure to cold temperatures was explained by endogenous disease, accident, suicide, and unidentified factors in 21, 120, 24, and 3 cases, respectively. Female patients were significantly more frequently exposed to cold conditions caused by suicide attempts, while a similar pattern was observed for accidents in males. Of the 120 cases involving accidents, 35 had several traumatic injuries presumably associated with falls, where the trauma was believed to be the main cause of immobility and subsequent exposure to cold air. Collection of clinical data was available in 153 cases, and 60 patients were considered to be CI‐positive. The details of CI were as follows: 23 cases reported by family members or caregivers, 15 cases of AD, 15 cases of unspecified dementia, 2 cases of dementia with Lewy body, 2 cases of mild cognitive impairment, 1 case of early‐onset AD, 1 case of frontotemporal dementia, and 1 case secondary to carbon monoxide poisoning. None of these individuals were bedridden. In particular, among patients aged 80 or more, 44 of the 62 patients (71%) had CI. Furthermore, among them, a premortem history of wandering was identified in 32 cases. Of these, 31 were presumed to have developed hypothermia while wandering, and 27 (87%) were found dead outdoors. The remaining four cases were found indoors, but all in buildings other than their homes. In addition, in one case, there was congenital intellectual disability, and the onset of the development of hypothermia occurred while wandering outdoors. Fourteen patients had a history of psychiatric visits, which were significantly more frequent in females and consistent with the higher number of suicide attempts in this group. In contrast, the number of cases with positive blood ethanol levels was significantly higher in male patients, which is consistent with the higher number of cases involving accidental exposure to cold temperatures in males. Furthermore, a clinical diagnosis of chronic alcoholism or a life situation strongly indicative thereof was identified only in male patients. Screening toxicology testing revealed positivity in 20 cases (benzodiazepines, tricyclic antidepressants, opioids, and barbituric acid in 16, 4, 3, and 1 cases, respectively). Nonetheless, detailed drug testing identified benzodiazepines, zolpidem tartrates, and tricyclic antidepressants in eight, three, and one cases, respectively, although none of them achieved lethal concentrations.

### 3.2 Neuropathological analysis results

**FIGURE 2:**
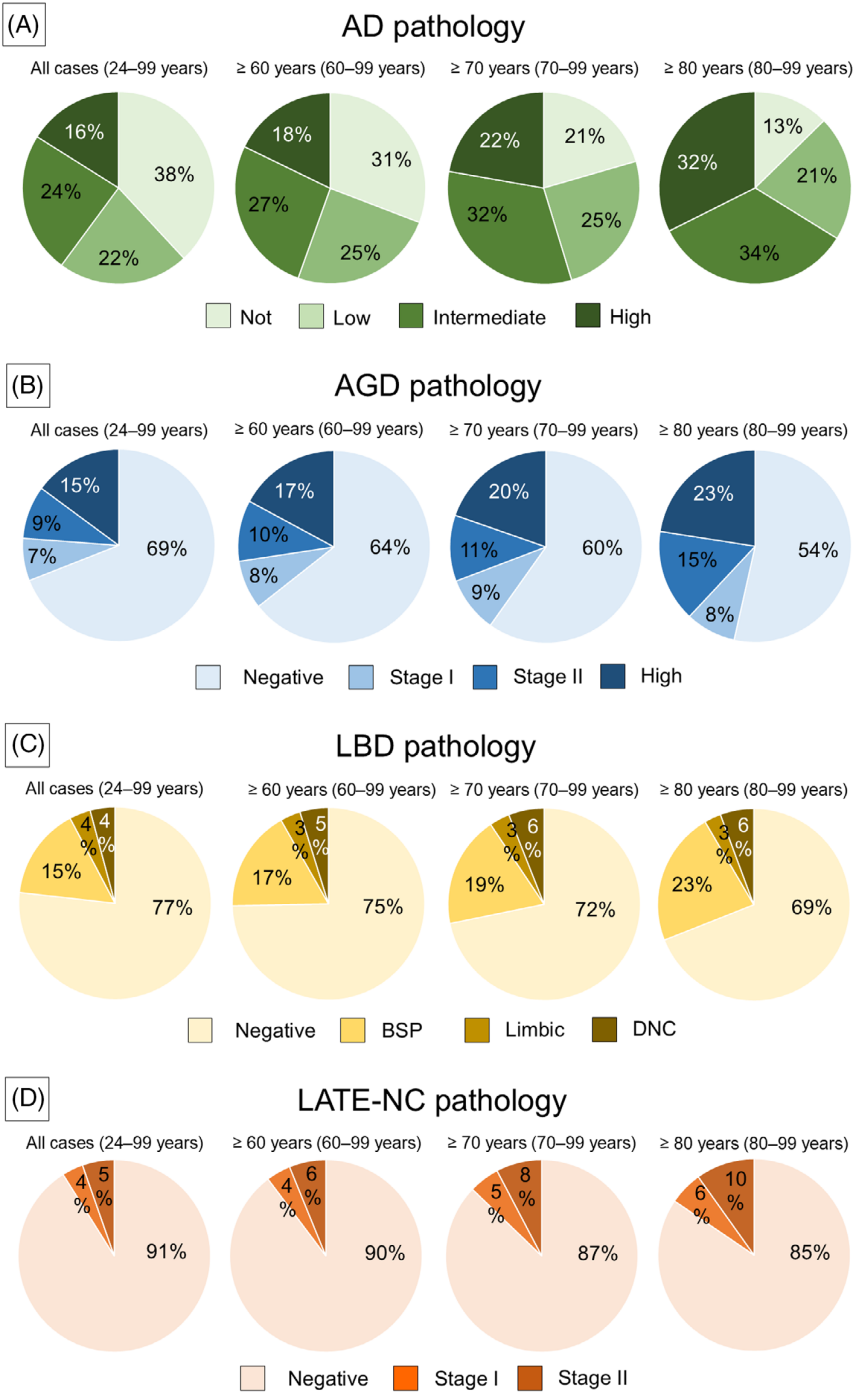
Prevalence of Alzheimer's disease (AD), argyrophilic grain disease (AGD), Lewy body disease (LBD), and limbic‐predominant age‐related TDP‐43 encephalopathy neuropathologic changes (LATE‐NC) pathology by age. Note that the frequency and histopathologic severity increased with age in all diseases, especially AD and AGD. BSP, brainstem‐predominant; DNC, diffuse neocortical; Int, intermediate level; Neg, negative.

Table 2 summarizes the outcomes of the neuropathological analysis of the cases, and Table S2 details them. The distribution of the frequency and severity by age for the four diseases with the largest number of patients, including AD, AGD, LBD, and LATE‐NC, is presented in Figure 2.

**TABLE 2:** Summary of neuropathological findings of all cases.

	All (*N* = 168)	Female (*N* = 75)	Male (*N* = 93)	*p‐*Value^a^
AD pathology (mean ± SD) [20]				
A score [0/1/2/3]	65/44/24/35 (1.2 ± 1.2)	27/20/11/17 (1.2 ± 1.2)	38/24/13/18 (1.1 ± 1.1)	0.49
B score [0/1/2/3]	10/50/68/40 (1.8 ± 0.9)	3/19/33/20 (1.9 ± 0.8)	7/31/35/20 (1.7 ± 0.9)	0.14
C score [0/1/2/3]	69/25/36/38 (1.3 ± 1.2)	27/13/21/14 (1.3 ± 1.1)	42/12/15/24 (1.2 ± 1.3)	0.67
AD level (Not/Low/Int/High; %)	64/37/40/27 (38/22/24/16)	26/17/20/12 (35/23/27/16)	38/20/20/15 (41/22/22/16)	NA
LBD pathology (mean ± SD) [22]				
0/1/2/3/4/5–6	129/20/1/5/6/7 (0.6 ± 1.4)	56/8/1/3/4/3 (0.7 ± 1.4)	73/12/0/2/2/4 (0.5 ± 1.3)	0.50
AGD pathology (mean ± SD) [24]				
0/1/2/3	116/12/15/25 (0.7 ± 1.1)	48/9/9/9 (0.7 ± 1.1)	68/3/6/16 (0.7 ± 1.2)	0.43
LATE‐NC pathology (mean ± SD) [27, 28]				
0/1/2/3	153/6/9/0 (0.1 ± 0.5)	68/1/6/0 (0.2 ± 0.6)	85/5/3/0 (0.1 ± 0.4)	0.80
Other neuropathology presumed to have contributed to exposure to cold conditions (%)				
PSP	11 (7)	6 (8)	5 (5)	0.54
CBD	2 (1)	1 (1)	1 (1)	1
Pick disease	1 (1)	1 (1)	0	0.45
Old infarction^b^	11 (7)	6 (8)	5 (5)	0.54
c‐SH	1 (1)	0	1 (1)	1
PMDN	1 (1)	0	1 (1)	1
Chronic CO toxicity	1 (1)	0	1 (1)	1
Alc‐related pathologies	5 (3)	0	5 (3)	0.07
MN‐related pathologies (Alc‐Neg)	2 (1)	0	2 (1)	0.5

Abbreviations: AD, Alzheimer's disease; AGD, argyrophilic grain disease; Alc, (chronic) alcoholism; CBD, corticobasal degeneration; CO, carbon monoxide; c‐SH, classic‐type superficial hemosiderosis; LATE‐NC, limbic‐predominant age‐related TDP‐43 encephalopathy neuropathologic changes; LBD, Lewy body disease; MN, malnutrition; Neg, negative; PMDN, primary melanosis of the dentate nucleus; PSP, progressive supranuclear palsy; SD, standard deviation.

^a^ Female versus male patients, evaluated using Fisher's exact test or Mann–Whitney *U* test.

^b^ Including cerebral, cerebellar, and lacunal infarction.

AD pathology was the most frequent finding, with 67 cases (40% of all cases) classified as an intermediate level or higher, as per the NIA‐AA guidelines. The frequency and severity of AD pathology increased with age, and approximately two‐thirds of patients aged 80 years or older had a pathology at an intermediate level or higher (Figure 2A). Notably, 16 of the 67 patients (24%) with this AD pathology exhibited no CI before death. AGD, LBD, and LATE‐NC pathology were found in 52, 39, and 15 cases, respectively (31%, 23%, and 9% of all cases, respectively) (Figure 2B–D). The frequency and pathological severity of these diseases tended to positively correlate with age, particularly in the case of AGD (Figure 2B), while no significant differences in severity were detected between the genders. Furthermore, the number of cases with CI was significantly higher in the outdoor hypothermia group than in the indoor group (*p* = 0.0498). In contrast, there were no significant differences in the frequencies of major NDDs, including AD, AGD, LBD, and LATE‐NC between the two groups (Table S3).

Other pathologies included PSP, old cerebral/cerebellar infarction, and malnutrition‐related pathology in 11, 11, and 7 cases, respectively. Notably, malnutrition‐related pathologies, both unrelated and related to chronic alcoholism, were detected only in male patients (*p* = 0.02). In addition, other neurological disorders, including corticobasal degeneration (CBD), Pick's disease, classic superficial hemosiderosis, primary melanosis of dentate nucleus, and a case of chronic carbon monoxide toxicity were identified.

### 3.3 Neuropathological findings in CI‐positive patients

**FIGURE 3:**
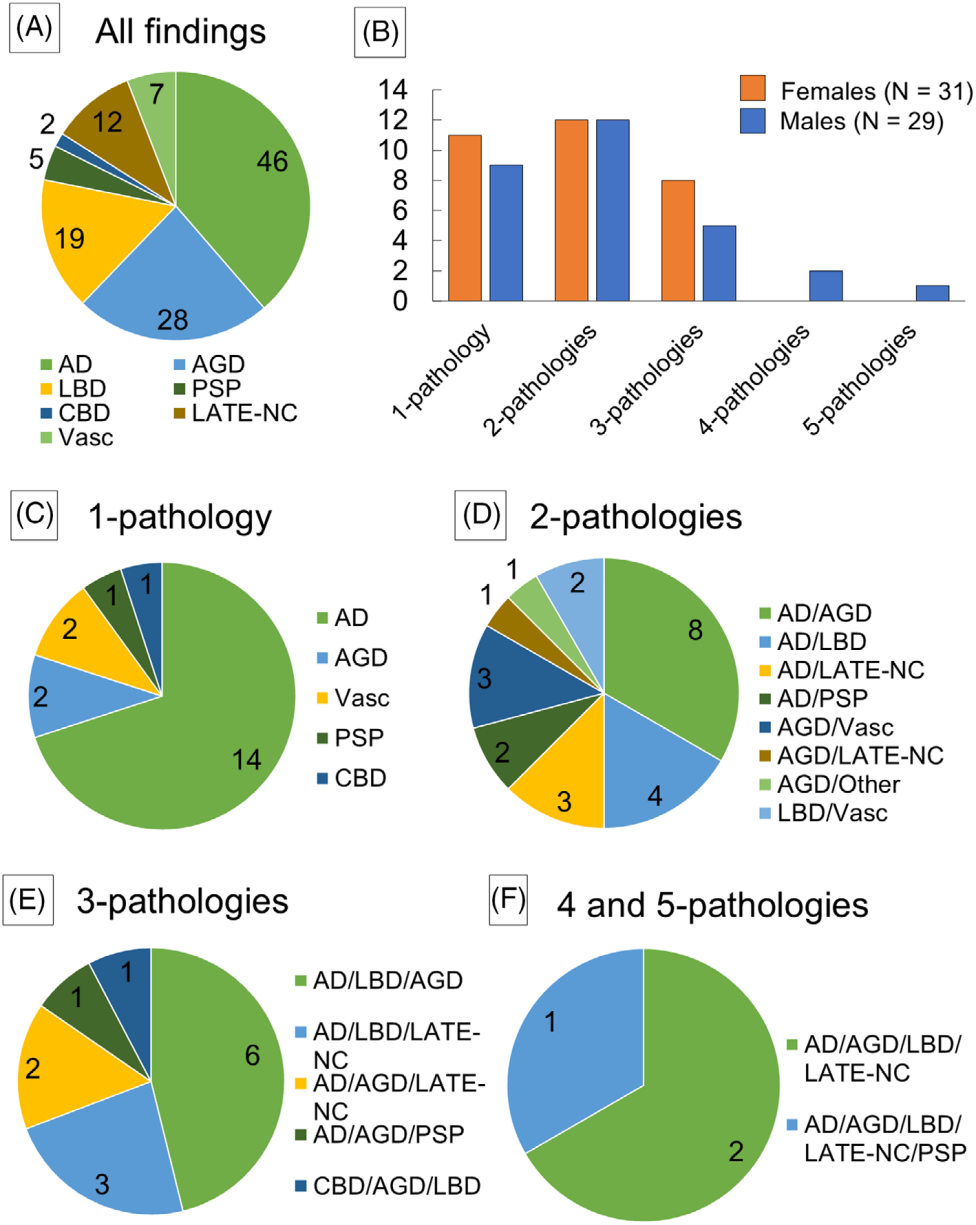
Neuropathological findings in patients with cognitive impairment (CI). (A) All neuropathological findings. (B) Frequency of combinations of neuropathological diseases. Frequency of CI‐causative diseases in groups with one (C), two (D), three (E), and four and five pathologies (F). CBD, corticobasal degeneration; PSP, progressive supranuclear palsy; Vasc, vascular dementia.

The results of the neuropathological analysis of the 60 cases are illustrated in Figure 3. Table S3 summarizes the neuropathological findings in all the cases, and Table S4 provides detailed results thereof. Among NDDs, AD was the most commonly found in 46 cases (77%, Intermediate‐level 22 cases; High‐level 24 cases), followed by AGD in 28 (47%), LBD in 19 (32%), and LATE‐NC in 12 cases (20%) (Figure 3A). The most common neuropathological condition accompanying CI was the combination of two pathologies, which was observed in 24 cases (40%), followed by single pathology in 20 (33%) and triple in 13 cases (22%) (Figure 3B). The majority of cases in which CI was attributed to a single pathology had AD (70%) (Figure 3C). In both the two‐ and three‐pathology groups, AD was also the most common complication and was found in 17 (63%) and 12 cases (92%), respectively (Figure 3D,E). Interestingly, two cases with four pathologies (AD/AGD/LBD/LATE‐NC) and one with five (AD/AGD/LBD/LATE‐NC/PSP) were observed (Figure 3F), all of which were males (representative findings from the latter case are presented in Figure S1). However, no significant differences were detected in the frequency or severity of neuropathological diseases between the gender groups.

We further evaluated 31 cases in which hypothermia developed while wandering. The results of the neuropathological analysis are shown in Figure S1, with the details of the findings outlined in Table S5. Although a higher proportion of cases with intermediate or greater AD pathology was observed in the wandering‐positive group (27 cases, 87%) compared with the wandering‐negative group (19 cases, 66%), the difference did not reach statistical significance (*p* = 0.07). In addition, no significant differences in the frequencies of AGD, LBD, or LATE‐NC were observed between the two groups.

### 3.4 Neuropathological findings in patients with multiple traumatic injuries associated with falls

**FIGURE 4:**
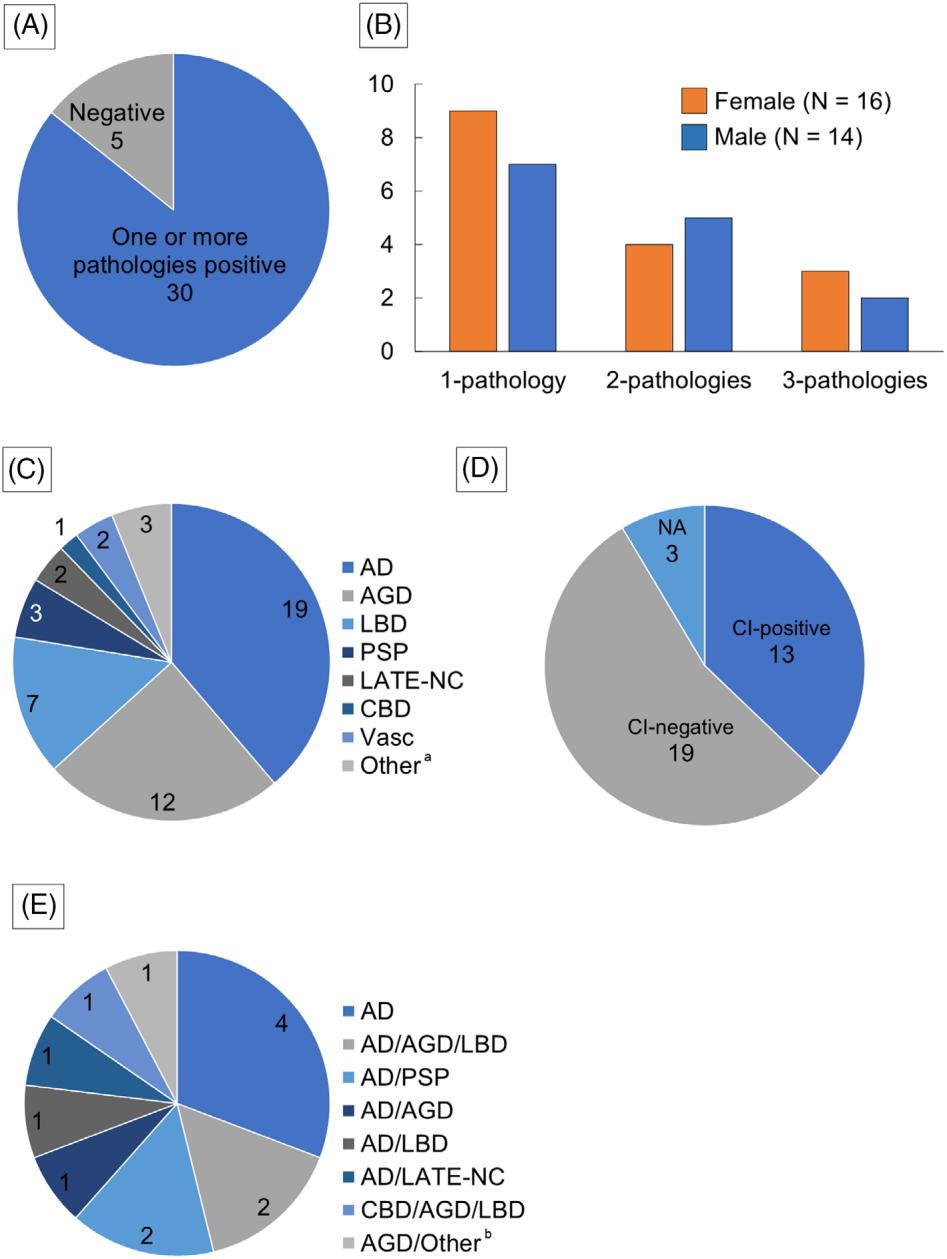
Neuropathological findings in individuals with multiple traumatic injuries related to falls. (A) Proportion of cases with or without neuropathological conditions. (B) Frequency of combinations of neuropathological diseases. (C) All neuropathological findings. (D) Proportion of cases with and without CI. (E) Proportion of causative diseases of CI. ^a^ Includes one case of classic‐type superficial hemosiderosis, one case of mild hemorrhage of the caudate nucleus and cerebral contusion, and one case of chronic carbon monoxide toxicity. ^b^ AGD and chronic carbon monoxide toxicity.

Table 3 summarizes the neuropathological findings in the 35 cases, Table S6 provides additional details, and the results are demonstrated in Figure 4. Traumatic injuries occurred more frequently outdoors than indoors, and fractures were observed in 27 cases. Furthermore, facial and/or head injuries—indicators of external force that may have caused concussion or loss of consciousness—were observed in 24 cases (69%). Neuropathological conditions were diagnosed in 30 of the 35 patients (86%) (Figure 4A). The most common manifestation was a single pathology in 16 cases (53%), followed by double (30%) and triple (17%) pathologies (Figure 4B). No cases involving more than three pathologies were identified in this group. Among the neuropathological conditions, AD (39%) was the most prevalent, followed by AGD (24%) and LBD (14%) (Figure 4C). In addition, two cases of PSP and one of CBD were identified. CI was revealed in 13 cases (Figure 4D), with four individuals presumed to have fallen while wandering. Among CI‐positive cases, nine (69%) had two or more neuropathological conditions, whereas only AD was implicated as a cause of CI in the single pathology cases (Figure 4E). We investigated potential differences in clinical information, complications, and the frequency of neurodegenerative diseases between the fall‐related trauma–positive group and the other cases. The results are summarized in Table 3. The fall‐positive group showed a slightly higher proportion of individuals aged ≥65 years and a tendency toward a higher frequency of intermediate or severe AD pathology, but these differences were not statistically significant.

**TABLE 3:** Summary of clinical and neuropathological findings in patients with several traumatic injuries associated with falls.

	Fall‐cases	Non‐fall cases	*p*‐Value^a^
Number of cases (F/M)	35 (19/16)	133 (56/77)	0.25
Mean age in years (range)	77.6 ± 8.5 (59–91)	73.0 ± 15.3 (24–99)	0.27
# of patients aged ≥65 years (%)	32 (91)	105 (79)	0.14
Outdoor/indoor (%)	25/10 (71/29)	96/37 (72/28)	1
Complications (%)			
Cognitive impairment	13 (41)^b^	47 (39)^c^	0.84
Wandering	4 (13)^b^	27 (22)^c^	0.32
Psychiatric visits^b^	4 (13)^b^	10 (8)^c^	0.49
Toxicology testing (%)			
Blood ethanol‐positive	2 (6)	14 (10)	0.74
Drug‐positive	5 (14)	16 (12)^d^	0.78
Neurodegenerative diseases (%)			
AD^e^	19 (54)	48 (36)	0.055
AGD	12 (34)	40 (30)	0.68
LBD	7 (20)	32 (24)	0.82
PSP	3 (9)	8 (6)	0.70
LATE‐NC	2 (6)	13 (10)	0.74
CBD	1 (3)	1 (1)	0.37

^a^ Fall versus non‐fall patients, evaluated using Fisher's exact test or Mann–Whitney *U* test.

^b^ Frequency in 32 cases (18 female and 14 male patients), in whom clinical information was available.

^c^ Frequency in 121 cases (50 female and 71 male patients), in whom clinical information was available.

^d^ Frequency in 131 cases (55 female and 76 male patients), in whom drug screening testing results were available.

^e^ Number of cases with an intermediate level or higher, as per the NIA‐AA guidelines.

### 3.5 Comparison of hypothermia‐related deaths and other causes of death in individuals aged 65 years or older

To further investigate the frequency and significance of NDDs in hypothermia‐related deaths, we compared these cases with non‐hypothermic deaths among individuals aged 65 years or older. A summary of the results is presented in Table 4. A total of 1154 cases were analyzed, with hypothermia cases comprising 11.7% of the cohort. The hypothermia group showed a significantly higher number of cases with CI and AD pathology of intermediate or higher levels compared to the non‐hypothermia group. The prevalence of LATE‐NC was also significantly higher in the hypothermia group, likely reflecting the association with more severe AD pathology [36]. In contrast, there were no significant differences between the two groups in the frequency of AGD or LBD pathology.

**TABLE 4:** Comparison of hypothermia‐related deaths and other causes of death in patients aged 65 years or older.

	Hypothermia (*N* = 135)	Other causes (*N* = 1019)	*p* Value^a^
Age; mean ± SD (range)	79.6 ± 7.9 (65–99)	78.7 ± 7.9 (65–101)	0.15
Sex (F/M)	66/69	464/555	0.46
Cognitive impairment‐positive (%)	59 (44)	185 (18)	**<0.01**
AD pathology‐positive (%)^b^	62 (46)	349 (34)	**<0.01**
AGD pathology‐positive (%)	49 (36)	324 (32)	0.33
LBD pathology‐positive (%)	36 (27)	220 (22)	0.19
LATE‐NC pathology‐positive (%)	15 (11)	59 (6)	**0.02**

*Note*: Boldface indicates statistically significant values at *p* < 0.05.

^a^ Hypothermia versus other causes, evaluated using Fisher's exact test or Mann–Whitney *U* test.

^b^ Number of cases with an intermediate level or higher, as per the NIA‐AA guidelines.

### 3.6 Cases involving patients aged under 65 years and with significant neuropathological disease

We found 8 out of 33 cases, including two with malnutrition‐related diseases, two with LBD (limbic stage), two with AD (one of which was familial) [37], one with AGD but without CI, and one with classic superficial hemosiderosis. Table 5 presents a summary of the clinical and neuropathological data of these cases. Notably, seven out of eight cases involved male patients. In six of these cases, there had been no history of neurological or psychiatric consultation prior to death, and no information was available regarding whether the patients had presented with these symptoms. However, in one case (Case 8), the patient had been confined at home and spent most of the time in a bed, which suggests the effects of LBD.

**TABLE 5:** Summary of clinical and neuropathological findings in patients aged less than 65 years.

#	Age	Sex	BW	Medical history	NV	PV	CI	M/D	S/D	B‐EtOH	Drug	Neuropathological condition	Other complications
1	36	M	1530	Chronic Alc	None	None	Neg	Suicide	OD	Pos	Neg	Pellagra encephalopathy	None
2	64	M	1508	MSA or c‐SH	Present	None	Neg	Illness	ID	Neg	TCA	c‐SH	Rib fracture
3	62	M	1556	Asthma	None	None	Neg	Accident	OD	Neg	BZO	LBD (Braak's LBD stage 4) [22]	None
4	59	M	1151	Anorexia?	None	None	Neg	Accident	ID	Neg	Neg	Chronic Wernicke's encephalopathy	MN (BMI 15.1)
5	60	M	1506	HT, HL, DM	None	None	Neg	Accident	OD	Pos	Neg	AGD (Saito's stage III) [24]	Fatty liver
6	59	M	1454	LD	None	None	Neg	Accident	OD	Neg	Neg	AD (A2B2C2, Intermediate level) [20]	GAC with liver metastasis, MN (BMI 15.4)
7	57	M	1494	Juvenile AD (familial)	Present	None	Pos	Accident	ID	Neg	Neg	AD (A3B3C3, High level) [20]	Fatty liver
8	44	F	1085	Uterine leiomyoma	None	None	Neg	Illness	ID	Neg	Neg	LBD (Braak's LBD stage 4) [22]	MN (BMI 14.6)

Abbreviations: BW, brain weight (g); BZO, benzodiazepines; CI, cognitive impairment; DM, diabetes mellitus; F, female; GAC, gastric adenocarcinoma; HL, hyperlipidemia; HT, hypertension; ID, indoor; M, male; M/D, manner of death; LD, liver dysfunction; MSA, multiple system atrophy; NV, history of neurology visits; OD, outdoor; PV, history of psychiatric visits; S/D, scene of the discovery; TCA, tricyclic antidepressants.

## 4 DISCUSSION

This study investigated the neuropathological conditions that contributed to the development of fatal hypothermia. To the best of our knowledge, this is the largest neuropathological study conducted in a forensic autopsy cohort. Our findings revealed that: (1) Fatal hypothermia predominantly occurred in older adults aged 65 years or more; (2) AD was the most common underlying NDD of fatal hypothermia, affecting 40% of all patients and approximately two‐thirds of those aged 80 years or more; (3) CI was often caused by mixed pathologies rather than a single pathology; (4) approximately half of the patients with CI developed hypothermia while wandering, and AD was present in most cases with this condition; (5) one or more neuropathological conditions were observed caused by fall‐related traumatic injuries; (6) Among individuals aged 65 years or older, the incidence of CI and AD was significantly higher in the hypothermic group than in the non‐hypothermic group; and (7) notable neuropathological diseases could also be identified among patients younger than 65 years.

Our findings indicate that fatal hypothermia was more likely to develop in older adults and that neuropathologic diseases were one of the underlying causes of the condition. Particularly, the presence of more than one pathology was assumed to be responsible for many of the CI‐positive and trauma cases associated with falls, rather than a single pathology. Recently, it has become increasingly recognized that combinations of one or more pathologies (mixed pathology) are frequently found in patients with NDDs [8, 38]. Furthermore, a growing body of evidence on mixed pathology suggests that it may lower the threshold for the development of CI [8, 39, 40, 41, 42, 43]. Thus, it is speculated that mixed pathology is likely to be the underlying mechanism of the condition that provokes many “unusual deaths” among older adults.

Notably, unlike in hospital‐based studies [2], most deaths caused by hypothermia in this study occurred outdoors. This may be explained by the finding that most cases of deaths related to wandering were detected outdoors. Specifically, in more than half of the CI‐positive cases in this study, patients were presumed to have developed hypothermia while wandering. These results are consistent with previous research showing that hypothermia is a common cause of death among individuals with dementia during episodes of wandering [44, 45]. AD is thought to be the most common underlying cause of wandering [46]. In this study, AD pathology at an intermediate level or higher was observed in most cases with wandering. Furthermore, the hypothermia‐related death group exhibited a significantly higher frequency of CI and AD pathology compared to the non‐hypothermia‐related death group. Thus, it is indicated that AD is a core component of the progression of this condition. As previously reported, outdoor deaths are more likely to happen in exposure to wet or strong windy conditions, causing affected patients to suffer fatal hypothermia within a short time and are thus not transported to the hospital, as they are already lifeless when found [3]. These findings emphasize the need to recognize the considerable danger of wandering in cold environments. Of note, no differences in neuropathological findings were observed between wandering‐positive and ‐negative patients with CI in this study. Understanding the causes of wandering in CI‐positive cases is essential for preventing hypothermia, and further research is warranted.

Although no significant differences were observed, the fall‐related trauma‐positive group showed a higher proportion of individuals aged ≥65 years and a trend toward a higher frequency of intermediate or greater AD pathology compared with the negative group. This is consistent with the report that both the incidence of falls and the severity of fall‐related complications rise steadily after age 60 [47]. Furthermore, in regions like ours, where heavy snowfall and frequent ground freezing occur, the risk of immobility from slip‐related falls during walking (including wandering) is increased, potentially contributing to the onset of fall‐related trauma. The presence of intermediate or severe AD pathology may further increase this risk. Collectively, these results highlight the need to recognize that falls in cold environments pose a risk of hypothermia in elderly individuals.

We have shown that NDDs may underlie hypothermia‐related deaths in older adults as well as those younger than 65 years. Notably, these conditions were not diagnosed when the patients were alive. Moreover, pathologies associated with chronic alcoholism and malnutrition were considered to be important background conditions for the development of hypothermia in this group. Thus, without an accurate neuropathological examination, the presence of disease might have been overlooked in these cases. In particular, we recommend comprehensive neuropathological examinations in hypothermia‐related deaths among individuals under 65 years of age when the reasons for failure to move away from cold environments are unclear, as such cases may reveal underlying pathological changes contributing to the condition. Currently, it remains unclear whether the prevalence of NDDs is higher in hypothermia cases than in deaths from other causes among individuals aged 65 years or younger, highlighting the need for further investigation.

Our study has limitations represented by the clinical characteristics of some patients, mainly the lack of severity in clinical manifestation and low consultation rates in the participating medical institution. Furthermore, this study did not examine the effects of some neuropathological conditions that affect cognitive function, including cerebral amyloid angiopathy [30], primary age‐related tauopathy [48], and age‐related tau astrogliopathy [49]. Moreover, we have not evaluated the genotypes that may pose a risk for the development of AD, including *APOE* [50]. A further study on these aspects is thus considered necessary in the future. In addition, as demonstrated in this study, forensic autopsy cohorts may be significantly influenced by climatic and geographic factors; therefore, caution is warranted when comparing these results with those from other studies. Interestingly, Byard et al. reported that elderly people with wandering dementia are at risk of death not only from hypothermia but also from hyperthermia [9], and further research based on detailed neuropathological examination is needed in this regard.

In conclusion, we demonstrate that various neuropathological conditions can provoke fatal hypothermia. AD was the most commonly found pathology, whereas CI was most frequently associated with a combination of multiple‐pathology diseases (mixed pathology). There should be an increased awareness of the dangers of dementia with wandering in cold environments. In addition, notable neuropathology was identified in some cases of patients younger than 65 years, many of whom had been undiagnosed prior to death. These findings highlight the need for comprehensive neuropathological examinations in forensic autopsy cases. Notably, the underlying causes of hypothermia may vary depending on cohort characteristics, particularly climate and geography. While forensic autopsy‐based research has inherent limitations, it offers valuable insights relevant to the target region and other areas with similar environmental conditions. Therefore, to establish effective countermeasures, the role of neuropathological diseases in the development of fatal hypothermia should be assessed and interpreted on a country‐by‐country or region‐by‐region basis.

## AUTHOR CONTRIBUTIONS

SI conceived and designed the study, conducted the neuropathological observations, and drafted the manuscript and figures; KY and RT provided the clinical information and reviewed the manuscript. All authors have read and approved the final version of the manuscript.

## FUNDING INFORMATION

This research was supported by the FIRSTBANK OF TOYAMA SCHOLARSHIP FOUNDATION RESEARCH GRANT awarded to S.I.

## CONFLICT OF INTEREST STATEMENT

The authors declare that they have no competing interests.

## ETHICS STATEMENT

All the autopsies, including neuropathological investigations, were performed with written consent from the legal authority under the criminal code. All experimental protocols, including the use of the pathological specimens obtained at autopsy for research upon anonymization, were approved by the official ethical institutional review board of the University of Toyama (R2020006). This study was performed in accordance with the ethical standards established by the 1964 Declaration of Helsinki.

## Supporting information


**Appendix S1:** Supporting information.

## Data Availability

The data that support the findings of this study are available on request from the corresponding author. The data are not publicly available due to privacy or ethical restrictions.
