# Supplementary material for: Alzheimer's disease and mixed pathologies as a hidden contributor to fatal hypothermia: A large‐scale forensic autopsy‐based study
Source: Brain Pathol. 2025 Nov 30;36(3):e70051. doi: 10.1111/bpa.70051 (PMC13052153; doi:10.1111/bpa.70051)
Supplement: Supplementary file 1 — Appendix S1: Supporting information. [file BPA-36-e70051-s001.docx]

**ONLINE SUPPLEMENTARY DATA**

Alzheimer’s disease and mixed pathologies as a hidden contributor to fatal hypothermia: A large-scale forensic autopsy-based study

Shojiro Ichimata^a^, Koji Yoshida^a^, Ryo Tanaka^b^

^a^ Department of Legal Medicine, Faculty of Medicine, University of Toyama, Toyama, Japan

^b^ Department of Neurology, Toyama University Hospital, Toyama, Japan

**List of Supplemental material**

Supplemental Table 7

Supplementary Figure 2

**Table S1.** All clinical information of the cases in this study.

|  | Age | Sex | BH (cm) | BW (g) | BMI | Brain (g) | M/D | S/D | PI | Bone fracture/cause | Cognitive  impairment/type | Wondering | Psychological  disorder | B-EtOH | Drug |
| --- | --- | --- | --- | --- | --- | --- | --- | --- | --- | --- | --- | --- | --- | --- | --- |
| 1 | 88 | M | 154 | 41.1 | 17.3 | 1221 | Acc | OD | 2 | Neg | Pos/CDUD | Pos | Neg | 0 | Neg |
| 2 | 63 | M | 166 | 66 | 24.0 | 1208 | Acc | OD | 5 | Neg | Neg | Neg | Neg | 0 | BZO, TCA |
| 3 | 87 | F | 147 | 33.7 | 15.6 | 1060 | Ill | OD | 5 | Neg | Neg | Neg | Neg | 0 | BZO |
| 4 | 70 | M | 157 | 50.1 | 20.3 | 1363 | Acc | ID | 1 | Neg | Neg | Neg | Schiz | 0 | Neg |
| 5 | 36 | M | 167 | 45.6 | 16.4 | 1530 | Sui | OD | 1 | Neg | Neg | Neg | Neg | 2.3 | Neg |
| 6 | 89 | F | 143 | 35.6 | 17.4 | 1336 | Ill | ID | 3 | RB/Unk | NA | NA | NA | 0 | Neg |
| 7 | 82 | M | 152 | 57.6 | 24.9 | 1313 | Acc | OD | 9 | Neg | Pos/FCS | Pos | Neg | 0.12 | Neg |
| 8 | 78 | M | 160 | 53.2 | 20.8 | 1351 | Acc | OD | 1 | RtHum/Fall | Pos/FCS | Neg | Neg | 0 | Neg |
| 9 | 82 | M | 167 | 54 | 19.4 | 1459 | Acc | OD | 10 | Neg | NA | NA | NA | 0 | Neg |
| 10 | 74 | M | 164 | 50 | 18.6 | 1368 | Ill | OD | 2 | RB/Fall | Neg | Neg | Neg | 0.44 | Neg |
| 11 | 84 | F | 150 | 37.2 | 16.5 | 1243 | Sui | OD | 1 | RB/CPR | Neg | Neg | Neg | 0 | Neg |
| 12 | 85 | M | 157 | 51 | 20.7 | 1396 | Ill | ID | 1 | ThV/Fall | Pos/FCS | Neg | Neg | 0 | Neg |
| 13 | 71 | M | 168 | 61.9 | 21.9 | 1386 | Acc | OD | 1 | Neg | Neg | Neg | Neg | 0 | Neg |
| 14 | 76 | M | 147 | 58 | 26.8 | 1340 | Ill | OD | 1 | Neg | NA | NA | NA | 1.912 | Neg |
| 15 | 64 | M | 169.5 | 63.3 | 22.0 | 1314 | Ill | ID | 6 | Neg | Neg | Neg | Neg | 0.867 | Neg |
| 16 | 70 | M | 169 | 52.3 | 18.3 | 1574 | Unk | ID | 2 | RB/CPR | Neg | Neg | Neg | 0 | Neg |
| 17 | 88 | F | 137 | 32.4 | 17.3 | 1170 | Ill | ID | 1 | RB/CPR | NA | NA | NA | 0 | Neg |
| 18 | 78 | F | 144 | 45.6 | 22.0 | 1337 | Acc | OD | 1 | Neg | Neg | Neg | Neg | 0 | Neg |
| 19 | 61 | F | 156 | 47.8 | 19.6 | 1304 | Acc | OD | 1 | LtHum/RtHum/Fall | Neg | Neg | Schiz | 0 | BZO |
| 20 | 32 | M | 176 | 63.3 | 20.4 | 1378 | Sui | ID | 3 | Neg | Neg | Neg | EatD/GID | 0 | BZO, BAR |
| 21 | 83 | M | 160 | 44.2 | 17.3 | 1472 | Acc | OD | 2 | Neg | NA | NA | NA | 0 | Neg |
| 22 | 84 | F | 142 | 41.6 | 20.6 | 1300 | Acc | OD | 3 | RB/Pel/Fall | Pos/MCI | Neg | Neg | 0 | Neg |
| 23 | 91 | F | 131 | 30.4 | 17.7 | 1233 | Acc | OD | 1 | Neg | NA | NA | NA | 0 | Neg |
| 24 | 64 | M | 172 | 70.9 | 24.0 | 1508 | Ill | ID | 3 | RB/Fall | Neg | Neg | Neg | 0 | TCA |
| 25 | 62 | M | 162 | 53.7 | 20.5 | 1556 | Acc | OD | 3 | Neg | Neg | Neg | Neg | 0 | BZO |
| 26 | 62 | F | 157 | 47.6 | 19.3 | 1466 | Acc | ID | 1 | Neg | Neg | Neg | Schiz | 0 | NA |
| 27 | 66 | M | 154 | 53.3 | 22.5 | 1373 | Ill | ID | 2 | RB/Ste/CPR | Neg | Neg | Neg | 0 | Neg |
| 28 | 75 | M | 154 | 51.6 | 21.8 | 1350 | Acc | OD | 1 | Neg | Neg | Neg | Neg | 0.6 | Neg |
| 29 | 82 | F | 145 | 40 | 19.0 | 1032 | Ill | ID | 2 | Neg | Neg | Neg | Neg | 0 | Neg |
| 30 | 62 | M | 165 | 50.8 | 18.7 | 1508 | Ill | OD | 4 | Neg | Neg | Neg | Neg | 0 | Neg |
| 31 | 65 | M | 174 | 55.5 | 18.3 | 1393 | Acc | OD | 1 | Neg | Pos/FTD | Pos | Neg | 0 | Neg |
| 32 | 78 | F | 150 | 34.4 | 15.3 | 1240 | Acc | OD | 2 | Neg | Pos/AD | Neg | Neg | 0 | Neg |
| 33 | 85 | F | 140 | 24.7 | 12.6 | 1144 | Sui | OD | 2 | Neg | Neg | Neg | Neg | 0 | Neg |
| 34 | 65 | M | 168 | 55.3 | 19.6 | 1395 | Acc | OD | 1 | Neg | Neg | Neg | Neg | 0 | Neg |
| 35 | 59 | M | 152 | 35 | 15.1 | 1151 | Acc | ID | 5 | Neg | Neg | Neg | Neg | 0 | Neg |
| 36 | 84 | M | 156 | 52.8 | 21.7 | 1265 | Acc | OD | 4 | Neg | Pos/FCS | Neg | Neg | 0 | Neg |
| 37 | 81 | F | 135 | 36.3 | 19.9 | 1177 | Acc | OD | 1 | Neg | Neg | Neg | Neg | 0 | Neg |
| 38 | 78 | F | 150 | 46.6 | 20.7 | 1161 | Acc | OD | 1 | RB/CPR | Pos/FCS | Neg | Neg | 0.678 | BZO |
| 39 | 76 | M | 177 | 59.8 | 19.1 | 1310 | Acc | OD | 1 | Neg | Neg | Neg | Neg | 0.399 | Zol |
| 40 | 99 | F | 149 | 33.5 | 15.1 | 1190 | Sui | OD | 1 | Neg | Pos/FCS | Neg | Neg | 0 | Neg |
| 41 | 84 | F | 140 | 30.3 | 15.5 | 1061 | Acc | ID | 10 | Neg | Pos/AD | Pos | Neg | 0 | Neg |
| 42 | 86 | F | 142 | 40.8 | 20.2 | 1082 | Sui | OD | 3 | RB/Ste/CPR | Pos/FCS | Neg | Neg | 0 | Neg |
| 43 | 91 | F | 149 | 40.7 | 18.3 | 1204 | Sui | OD | 2 | Cla/RB/Fall | Pos/FCS | Neg | Neg | 0 | Neg |
| 44 | 85 | M | 154 | 58.3 | 24.6 | 1280 | Acc | OD | 2 | LtFem/RtFem/Fall | NA | NA | NA | 0 | Neg |
| 45 | 85 | M | 173 | 51.4 | 17.2 | 1554 | Acc | OD | 3 | RB/CPR | Pos/FCS | Neg | Neg | 0 | Neg |
| 46 | 78 | F | 149 | 45.6 | 20.5 | 1202 | Acc | OD | 3 | RB/Fall | Neg | Neg | Neg | 0 | Neg |
| 47 | 80 | M | 169 | 47.6 | 16.7 | 1382 | Acc | ID | 4 | Neg | Pos/FCS | Neg | Neg | 0 | Neg |
| 48 | 71 | M | 155 | 60 | 25.0 | 1522 | Ill | ID | 5 | Neg | Neg | Neg | Neg | 0 | Neg |
| 49 | 66 | M | 155 | 58.7 | 24.4 | 1582 | Acc | OD | 2 | Neg | Neg | Neg | Neg | 0.879 | Neg |
| 50 | 32 | F | 159 | 57.5 | 22.7 | 1224 | Sui | OD | 2 | Neg | Neg | Neg | M-dep | 0 | BZO, TCA |
| 51 | 41 | F | 165 | 33.5 | 12.3 | 1390 | Ill | ID | 21 | Neg | NA | NA | NA | 0 | Neg |
| 52 | 72 | F | 154 | 28.3 | 11.9 | 1140 | Ill | ID | 21 | Neg | NA | NA | NA | 0 | Neg |
| 53 | 89 | F | 142 | 32.5 | 16.1 | 1355 | Acc | OD | 5 | Neg | Neg | Neg | Neg | 0 | Neg |
| 54 | 89 | M | 170 | 52.1 | 18.0 | 1540 | Acc | OD | 2 | Neg | Neg | Neg | Neg | 0 | Neg |
| 55 | 91 | F | 151 | 43.7 | 19.2 | 1260 | Acc | OD | 4 | Neg | Pos/FCS | Pos | Neg | 0 | Neg |
| 56 | 76 | F | 141 | 46.1 | 23.2 | 1063 | Acc | OD | 4 | Neg | Neg | Neg | Neg | 0 | Neg |
| 57 | 86 | M | 154 | 48.1 | 20.3 | 1169 | Acc | OD | 2 | RtFem/LuV/Fall | Pos/FCS | Neg | Neg | 0 | Neg |
| 58 | 68 | F | 153 | 35.6 | 15.2 | 1218 | Acc | OD | 1 | Neg | Neg | Neg | Neg | 0 | Neg |
| 59 | 75 | M | 163.4 | 55.5 | 20.8 | 1272 | Acc | OD | 3 | Neg | Pos/AD | Pos | Neg | 0 | Neg |
| 60 | 75 | F | 146 | 37.9 | 17.8 | 1108 | Acc | OD | 3 | RB/Ste/CPR | Pos/CDUD | Neg | Neg | 0 | Neg |
| 61 | 48 | F | 151 | 44.8 | 19.6 | 1343 | Sui | OD | 6 | Neg | Neg | Neg | Dep | 0 | Neg |
| 62 | 68 | M | 181 | 71 | 21.7 | 1473 | Acc | OD | 4 | Neg | Neg | Neg | Neg | 0 | Neg |
| 63 | 53 | M | 165 | 55.5 | 20.4 | 1431 | Acc | OD | 5 | Neg | Neg | Neg | Neg | 0 | Neg |
| 64 | 89 | M | 155 | 43.9 | 18.3 | 1432 | Acc | OD | 2 | Neg | Pos/MCI | Pos | Neg | 0 | Neg |
| 65 | 85 | M | 160 | 40.5 | 15.8 | 1263 | Acc | OD | 2 | RB/CPR | Pos/AD | Pos | Neg | 0 | Neg |
| 66 | 73 | M | 167 | 48.2 | 17.3 | 1360 | Acc | ID | 4 | Neg | Neg | Neg | Neg | 0 | Neg |
| 67 | 66 | M | 153 | 34.1 | 14.6 | 1414 | Acc | OD | 3 | Neg | Pos/CCOT | Neg | Neg | 0 | Neg |
| 68 | 67 | F | 154 | 46.1 | 19.4 | 1313 | Sui | OD | 3 | Neg | Neg | Neg | Schiz | 0 | Neg |
| 69 | 65 | M | 166 | 47.7 | 17.3 | 1464 | Sui | OD | 6 | Neg | Neg | Neg | Neg | 0 | BZO |
| 70 | 74 | F | 161 | 32.7 | 12.6 | 1116 | Acc | ID | 5 | Neg | Neg | Neg | Schiz | 0 | Neg |
| 71 | 65 | M | 160 | 54.7 | 21.4 | 1381 | Acc | OD | 2 | CeV/Fall | Neg | Neg | Neg | 2.194 | Neg |
| 72 | 52 | M | 184 | 76.5 | 22.6 | 1780 | Sui | OD | 21 | Neg | Neg | Neg | Dep | 0 | BZO |
| 73 | 93 | F | 147 | 29.2 | 13.5 | 1161 | Ill | ID | 2 | Neg | Pos/CDUD | Pos | Neg | 0 | Neg |
| 74 | 56 | F | 162 | 54.4 | 20.7 | 1217 | Sui | OD | 1 | Neg | Neg | Neg | Neg | 0 | Neg |
| 75 | 80 | F | 178 | 72.8 | 23.0 | 1586 | Sui | OD | 1 | CeV/RB/Fall/CPR | Pos/FCS | Neg | Neg | 0 | BZO |
| 76 | 60 | M | 170 | 68.3 | 23.6 | 1506 | Acc | OD | 2 | Neg | Neg | Neg | Neg | 2.268 | Neg |
| 77 | 83 | M | 158.8 | 48.9 | 19.4 | 1418 | Acc | OD | 3 | Neg | Neg | Neg | Neg | 0 | Neg |
| 78 | 86 | F | 146 | 37 | 17.4 | 1101 | Acc | OD | 1 | Neg | Pos/CDUD | Neg | Neg | 0 | Neg |
| 79 | 69 | M | 161 | 56.5 | 21.8 | 1210 | Acc | OD | 1 | Neg | Neg | Neg | Neg | 2.504 | Neg |
| 80 | 64 | M | 168 | 56.7 | 20.1 | 1371 | Acc | ID | 3 | Neg | Neg | Neg | Neg | 0 | Neg |
| 81 | 83 | M | 151 | 61.8 | 27.1 | 1206 | Acc | OD | 2 | Neg | Pos/AD | Pos | Neg | 0 | NA |
| 82 | 89 | F | 154 | 38.1 | 16.1 | 1273 | Acc | OD | 5 | Neg | Neg | Neg | Neg | 0 | BZO |
| 83 | 70 | F | 158 | 41.7 | 16.7 | 1262 | Sui | OD | 2 | Neg | Neg | Neg | Neg | 0 | Neg |
| 84 | 81 | M | 163 | 68 | 25.6 | 1216 | Acc | OD | 3 | Neg | Pos/AD | Pos | Neg | 0 | Neg |
| 85 | 71 | M | 154 | 41.3 | 17.4 | 1244 | Ill | ID | 5 | Neg | Neg | Neg | Neg | 0 | Neg |
| 86 | 73 | F | 145 | 46.2 | 22.0 | 1288 | Acc | ID | 1 | Neg | Neg | Neg | Neg | 0 | Neg |
| 87 | 83 | M | 151 | 38.9 | 17.1 | 1459 | Acc | ID | 1 | LuV/Fall | Pos/FCS | Neg | Neg | 0 | Neg |
| 88 | 55 | M | 167 | 62.1 | 22.3 | 1330 | Acc | OD | 13 | Neg | NA | NA | NA | 0 | Neg |
| 89 | 55 | F | 162 | 40.2 | 15.3 | 1324 | Sui | OD | 1 | Neg | Neg | Neg | Dep | 0 | Neg |
| 90 | 76 | M | 164 | 57.9 | 21.5 | 1542 | Acc | OD | 5 | Neg | Pos/FCS | Pos | Neg | 0 | Neg |
| 91 | 71 | F | 150 | 48.4 | 21.5 | 1276 | Sui | OD | 3 | ThV/Fall | Neg | Neg | Dep | 0 | BZO |
| 92 | 82 | F | 153 | 46.2 | 19.7 | 1129 | Acc | OD | 3 | Neg | Pos/AD | Pos | Neg | 0 | Neg |
| 93 | 41 | M | 170 | 56.1 | 19.4 | 1449 | Ill | OD | 4 | Neg | Neg | Neg | Neg | 0 | Neg |
| 94 | 65 | M | 164 | 48.2 | 17.9 | 1534 | Acc | OD | 2 | Neg | Neg | Neg | Neg | 0 | Neg |
| 95 | 88 | F | 143 | 35.2 | 17.2 | 1057 | Acc | OD | 1 | Neg | Neg | Neg | Neg | 0 | Neg |
| 96 | 91 | F | 155 | 34.7 | 14.4 | 1263 | Acc | ID | 1 | Neg | Pos/FCS | Neg | Neg | 0 | Neg |
| 97 | 68 | M | 155 | 54.3 | 22.6 | 1448 | Acc | OD | 1 | Neg | Neg | Neg | Neg | 2.8 | Neg |
| 98 | 78 | M | 164 | 49.3 | 18.3 | 1502 | Acc | OD | 3 | Neg | Neg | Neg | Neg | 0 | BZO |
| 99 | 87 | M | 157 | 50 | 20.3 | 1224 | Acc | OD | 2 | RB/CPR | Pos/CDUD | Pos | Neg | 0 | Neg |
| 100 | 67 | M | 166 | 56.8 | 20.6 | 1456 | Acc | OD | 3 | CeV/Fall | Neg | Neg | Neg | 1 | Neg |
| 101 | 39 | M | 175 | 63.6 | 20.8 | 1630 | Acc | ID | 5 | Neg | Neg | Neg | Neg | 3.5 | Neg |
| 102 | 85 | F | 155 | 34.1 | 14.2 | 1312 | Unk | ID | 3 | Neg | Neg | Neg | Neg | 0 | Neg |
| 103 | 84 | F | 150 | 54.8 | 24.4 | 1114 | Unk | OD | 1 | LtHum/RB/Fall/CPR | Pos/AD | Neg | Neg | 0 | Neg |
| 104 | 59 | M | 166 | 42.4 | 15.4 | 1454 | Acc | OD | 2 | CeV/Fall | Neg | Neg | Neg | 0 | Neg |
| 105 | 69 | F | 146 | 50.7 | 23.8 | 1300 | Acc | OD | 5 | CrB/ThV/Fall | Neg | Neg | Neg | 0 | Neg |
| 106 | 32 | M | 181 | 45.6 | 13.9 | 1480 | Sui | OD | 5 | Neg | NA | NA | NA | 0 | Neg |
| 107 | 81 | F | 147 | 43.6 | 20.2 | 1167 | Sui | OD | 2 | Neg | Neg | Neg | Neg | 0 | Neg |
| 108 | 80 | F | 146 | 45.9 | 21.5 | 1284 | Acc | ID | 4 | RtFem/Fall | Neg | Neg | Neg | 0 | Neg |
| 109 | 91 | F | 152 | 32.2 | 13.9 | 1116 | Acc | ID | 1 | LtFem/Fall | NA | NA | NA | 0 | Neg |
| 110 | 93 | F | 133 | 36.2 | 20.5 | 1162 | Acc | OD | 2 | Neg | Pos/CDUD | Pos | Neg | 0 | Neg |
| 111 | 75 | M | 173 | 68.1 | 22.8 | 1350 | Acc | OD | 2 | Neg | Neg | Neg | Neg | 0 | Neg |
| 112 | 82 | M | 160 | 53.1 | 20.7 | 1467 | Sui | OD | 3 | Neg | Neg | Neg | Neg | 0 | Neg |
| 113 | 24 | M | 177 | 57.9 | 18.5 | 1466 | Sui | OD | 7 | Neg | Neg | Neg | Neg | 0 | Neg |
| 114 | 82 | M | 162 | 50.8 | 19.4 | 1377 | Acc | OD | 2 | RB/Ste/CPR | NA | NA | NA | 0 | Neg |
| 115 | 85 | F | 143 | 42.1 | 20.6 | 1152 | Acc | OD | 18 | Neg | Pos/AD | Pos | Neg | 0 | Neg |
| 116 | 76 | F | 147 | 49.9 | 23.1 | 1243 | Acc | OD | 2 | FacB/Fall | Pos/DLB | Pos | Neg | 0 | Neg |
| 117 | 90 | F | 151 | 39.1 | 17.1 | 1235 | Ill | ID | 2 | Neg | NA | NA | NA | 0 | Neg |
| 118 | 86 | M | 149 | 36.5 | 16.4 | 1340 | Acc | OD | 2 | Neg | Pos/CDUD | Neg | Neg | 0 | Neg |
| 119 | 45 | M | 157 | 53.2 | 21.6 | 1580 | Acc | OD | 4 | Neg | Neg | Neg | Neg | 0 | Neg |
| 120 | 72 | F | 147 | 61 | 28.2 | 1152 | Acc | OD | 2 | Neg | Pos/CDUD | Neg | Neg | 0 | Neg |
| 121 | 75 | M | 174 | 57.1 | 18.9 | 1486 | Acc | ID | 6 | RB/Fall | Pos/CDUD | Pos | Neg | 0 | Neg |
| 122 | 86 | F | 145 | 32.3 | 15.4 | 1275 | Acc | ID | 6 | Neg | Pos/CDUD | Pos | Neg | 0 | Neg |
| 123 | 82 | F | 141 | 41.7 | 21.0 | 1263 | Acc | OD | 2 | RB/Ste/CPR | Pos/DLB | Neg | Neg | 0 | Neg |
| 124 | 90 | M | 160 | 54.1 | 21.1 | 1552 | Acc | OD | 4 | Neg | Neg | Neg | Neg | 0 | BZO |
| 125 | 57 | M | 162 | 64.8 | 24.7 | 1494 | Acc | ID | 3 | Neg | Pos/EOAD | Pos | Neg | 0 | Neg |
| 126 | 81 | M | 153 | 39.9 | 17.0 | 1339 | Acc | OD | 4 | Neg | IntD | Pos | Neg | 0 | Neg |
| 127 | 77 | F | 143 | 41.3 | 20.2 | 1196 | Acc | OD | 1 | CeV/Fall | Neg | Neg | Neg | 0 | Neg |
| 128 | 89 | M | 158 | 44.2 | 17.7 | 1322 | Acc | OD | 4 | RB/CPR | Pos/CDUD | Pos | Neg | 0 | Neg |
| 129 | 85 | F | 145 | 52.8 | 25.1 | 1337 | Acc | ID | 3 | Neg | Pos/AD | Neg | Neg | 0 | Neg |
| 130 | 79 | F | 153 | 38.5 | 16.4 | 1337 | Sui | OD | 3 | RtHum/LuV/Fall | Neg | Neg | Dep | 0 | BZO |
| 131 | 35 | M | 174 | 65.3 | 21.6 | 1598 | Acc | OD | 3 | Neg | Neg | Neg | Neg | 0 | Neg |
| 132 | 73 | F | 150 | 40 | 17.8 | 1331 | Acc | OD | 6 | Neg | Neg | Neg | Neg | 0 | Neg |
| 133 | 75 | F | 155 | 43.7 | 18.2 | 1246 | Acc | ID | 5 | Neg | Neg | Neg | Neg | 0 | Zol |
| 134 | 83 | M | 165 | 56.9 | 20.9 | 1329 | Acc | OD | 3 | Neg | Neg | Neg | Neg | 0 | Neg |
| 135 | 61 | M | 165 | 56.5 | 20.8 | 1272 | Acc | ID | 2 | Neg | Neg | Neg | Neg | 0 | Neg |
| 136 | 75 | M | 164 | 54.1 | 20.1 | 1298 | Acc | ID | 5 | Neg | Neg | Neg | Neg | 0 | Neg |
| 137 | 72 | M | 165 | 57 | 20.9 | 1426 | Acc | ID | 3 | Neg | Neg | Neg | Neg | 0 | Neg |
| 138 | 88 | F | 155 | 51.8 | 21.6 | 1255 | Acc | OD | 3 | CeV/Ste/RB/Fall/CRP | Pos/AD | Pos | Neg | 0 | Neg |
| 139 | 86 | M | 149 | 29.5 | 13.3 | 1212 | Acc | ID | 4 | RB/Ste/CPR | Pos/CDUD | Neg | Neg | 0 | Neg |
| 140 | 69 | F | 157 | 42.9 | 17.4 | 1120 | Sui | OD | 3 | Neg | Neg | Neg | Neg | 0 | Neg |
| 141 | 79 | F | 151 | 56 | 24.6 | 1226 | Acc | OD | 5 | Neg | Pos/FCS | Pos | Neg | 0 | Neg |
| 142 | 69 | M | 173 | 66.5 | 22.2 | 1406 | Acc | OD | 1 | Neg | Neg | Neg | Neg | 1.4 | OPI |
| 143 | 74 | M | 175 | 36.8 | 12.0 | 1502 | Acc | OD | 3 | Neg | Neg | Neg | Neg | 0 | Neg |
| 144 | 63 | F | 151 | 44.3 | 19.4 | 1488 | Acc | OD | 6 | Neg | Neg | Neg | Neg | 0 | Neg |
| 145 | 92 | M | 162 | 46.1 | 17.6 | 1198 | Acc | OD | 6 | Neg | Pos/FCS | Pos | Neg | 0 | Neg |
| 146 | 65 | M | 159 | 47.6 | 18.8 | 1532 | Acc | OD | 4 | Neg | Neg | Neg | Neg | 0 | Neg |
| 147 | 72 | F | 160 | 53.5 | 20.9 | 1330 | Sui | OD | 5 | RB/Ste/Fall | Neg | Neg | M-ep | 0 | Neg |
| 148 | 86 | M | 156 | 38.8 | 15.9 | 1230 | Acc | OD | 2 | Neg | Pos/CDUD | Neg | Neg | 0 | Neg |
| 149 | 90 | F | 145 | 40.8 | 19.4 | 1126 | Acc | OD | 14 | Neg | Pos/AD | Pos | Neg | 0 | Neg |
| 150 | 75 | F | 151 | 51.3 | 22.5 | 1300 | Acc | OD | 5 | Neg | Pos/FCS | Pos | Neg | 0 | Neg |
| 151 | 72 | M | 168 | 68.7 | 24.3 | 1468 | Acc | ID | 4 | CrB/Fall | Neg | Neg | Neg | 0 | Neg |
| 152 | 52 | M | 176 | 31.8 | 10.3 | 1564 | Ill | ID | 1 | Neg | NA | NA | NA | 0 | Neg |
| 153 | 78 | M | 164 | 48.4 | 18.0 | 1235 | Acc | OD | 5 | Neg | Pos/CDUD | Neg | Neg | 0 | Neg |
| 154 | 84 | M | 166 | 43.7 | 15.9 | 1175 | Acc | OD | 3 | Neg | Pos/FCS | Pos | Neg | 0 | Neg |
| 155 | 87 | M | 155 | 44.8 | 18.6 | 1349 | Acc | OD | 5 | Neg | Pos/AD | Pos | Neg | 0 | Neg |
| 156 | 86 | F | 138 | 28.9 | 15.2 | 1189 | Acc | OD | 5 | Neg | Pos/FCS | Pos | Neg | 0 | Neg |
| 157 | 92 | F | 143 | 42.6 | 20.8 | 1050 | Acc | OD | 2 | RB/Ste/CPR | Pos/CDUD | Pos | Neg | 0 | Neg |
| 158 | 55 | F | 150 | 47.4 | 21.1 | 1352 | Acc | ID | 4 | Neg | Neg | Neg | Schiz | 0 | BZO |
| 159 | 81 | F | 150 | 48.8 | 21.7 | 1143 | Acc | OD | 9 | Neg | Pos/AD | Pos | Neg | 0 | TCA |
| 160 | 76 | F | 151 | 40.4 | 17.7 | 1295 | Acc | OD | 3 | Neg | Neg | Neg | Neg | 0 | Neg |
| 161 | 76 | M | 182 | 55 | 16.6 | 1550 | Acc | ID | 4 | Neg | Neg | Neg | Neg | 0 | Neg |
| 162 | 79 | M | 160 | 47.1 | 18.4 | 1246 | Acc | OD | 2 | Neg | Pos/FCS | Pos | Neg | 0 | Neg |
| 163 | 90 | F | 137 | 40.8 | 21.7 | 1258 | Acc | OD | 1 | RB/Ste/CPR | Pos/FCS | Neg | Neg | 0 | Neg |
| 164 | 84 | F | 147 | 38.4 | 17.8 | 1159 | Acc | ID | 3 | Neg | Pos/AD | Neg | Neg | 0 | Neg |
| 165 | 73 | M | 159 | 63.8 | 25.2 | 1234 | Ill | ID | 2 | Neg | Neg | Neg | Neg | 0 | Zol |
| 166 | 85 | M | 157 | 56.3 | 22.8 | 1249 | Acc | ID | 2 | LuV/Fall | Neg | Neg | Neg | 0 | Neg |
| 167 | 74 | M | 151 | 47.5 | 20.8 | 1306 | Acc | OD | 1 | Neg | Neg | Neg | Neg | 0.61 | Neg |
| 168 | 44 | F | 153 | 34.2 | 14.6 | 1085 | Ill | ID | 1 | Neg | Neg | Neg | Neg | 0 | Neg |

**Abbreviations:** Acc, accident; AD, Alzheimer’s disease; BAR, barbituric acid; B-EtOH, blood ethanol concentration (mg/mL); BH, body height; BW, body weight; BZO, benzodiazepine; CCOT, chronic carbon monoxide toxicity; CDUD, clinical diagnosis of unspecified dementia; CeV, cervical vertebra; Cla, clavicle; CPR, cardiopulmonary resuscitation; CI, cognitive impairment; CrB, cranial bone; Dep, depression; DLB, dementia with Lewy body; EatD, eating disorder; EOAD, early-onset AD; F, female; FacB, facial bone; FCS, Families and/or caregivers' statements; Fem, femur; FTD, frontotemporal dementia; GID, gender identity disorder; Hum, humerus; ID, indoor; Ill, illness; IntD, intellectual disability; Lt, left; LuV, lumber vertebra; M, male; MCI, mild cognitive impairment; M/D, manner of death; M-dep, manic-depression; NA, not available; Neg, Neg; OD, outdoor; OPI, opioid; Pel, pelvis; PI, postmortem interval; Pos, positive; RB, rib bone; Rt, right; Schiz, schizophrenia; S/D, scene of the discovery; Ste, sternum; Sui, suicide; TCA, tricyclic antidepressant; ThV, thoracic vertebrae; Unk, unknown; Zol, zolpidem tartrate

**Table S2.** All neuropathological findings of the cases in this study.

|  | A | B | C | AD level | AGD | LBD | LATE-NC | PSP | Vascular | Other findings |
| --- | --- | --- | --- | --- | --- | --- | --- | --- | --- | --- |
| 1 | 3 | 3 | 3 | High | 0 | 5 | 0 | Neg | Neg |  |
| 2 | 0 | 1 | 0 | Not | 0 | 0 | 0 | Neg | Neg |  |
| 3 | 0 | 0 | 0 | Not | 0 | 0 | 0 | Neg | Neg |  |
| 4 | 2 | 2 | 3 | Int | 0 | 0 | 0 | Neg | Neg |  |
| 5 | 0 | 0 | 0 | Not | 0 | 0 | 0 | Neg | Neg | Pellagra encephalopathy (Alc) |
| 6 | 0 | 1 | 0 | Not | 3 | 3 | 1 | Neg | Neg |  |
| 7 | 1 | 2 | 2 | Int | 0 | 0 | 0 | Neg | Neg |  |
| 8 | 1 | 3 | 2 | Int | 0 | 0 | 0 | Pos | Neg |  |
| 9 | 2 | 2 | 3 | Int | 0 | 0 | 0 | Neg | Neg |  |
| 10 | 1 | 1 | 2 | Low | 3 | 0 | 0 | Neg | Neg | Pellagra encephalopathy (Alc) |
| 11 | 1 | 2 | 2 | Int | 1 | 0 | 0 | Neg | Neg |  |
| 12 | 3 | 3 | 3 | High | 0 | 0 | 0 | Pos | Neg |  |
| 13 | 0 | 2 | 0 | Not | 0 | 6 | 0 | Neg | Neg |  |
| 14 | 1 | 1 | 1 | Low | 0 | 3 | 0 | Neg | Neg |  |
| 15 | 0 | 1 | 0 | Not | 0 | 0 | 0 | Neg | Neg |  |
| 16 | 1 | 1 | 1 | Low | 0 | 0 | 0 | Neg | Pos |  |
| 17 | 1 | 2 | 1 | Low | 2 | 0 | 0 | Neg | Pos |  |
| 18 | 1 | 2 | 2 | Int | 1 | 0 | 0 | Neg | Neg |  |
| 19 | 0 | 1 | 0 | Not | 0 | 0 | 0 | Neg | Neg |  |
| 20 | 0 | 0 | 0 | Not | 0 | 0 | 0 | Neg | Neg |  |
| 21 | 2 | 2 | 3 | Int | 0 | 0 | 0 | Neg | Neg |  |
| 22 | 3 | 3 | 3 | High | 0 | 0 | 0 | Neg | Neg |  |
| 23 | 0 | 2 | 0 | Not | 3 | 2 | 1 | Neg | Pos |  |
| 24 | 0 | 1 | 0 | Not | 0 | 0 | 0 | Neg | Neg | Superficial hemosiderosis |
| 25 | 0 | 2 | 0 | Not | 0 | 4 | 0 | Neg | Neg |  |
| 26 | 0 | 1 | 0 | Not | 0 | 0 | 0 | Neg | Neg | Fahr disease |
| 27 | 1 | 1 | 2 | Low | 0 | 0 | 0 | Neg | Neg | Cerebellar degeneration (Alc) |
| 28 | 0 | 2 | 0 | Not | 3 | 0 | 0 | Neg | Pos |  |
| 29 | 1 | 1 | 1 | Low | 1 | 0 | 0 | Neg | Neg |  |
| 30 | 0 | 1 | 0 | Not | 0 | 0 | 0 | Neg | Pos | Old subdural hemorrhage |
| 31 | 0 | 3 | 0 | Not | 0 | 0 | 0 | Neg | Neg | Corticobasal degeneration |
| 32 | 3 | 3 | 3 | High | 0 | 0 | 1 | Neg | Neg |  |
| 33 | 2 | 2 | 3 | Int | 0 | 0 | 0 | Neg | Neg |  |
| 34 | 1 | 1 | 1 | Low | 0 | 0 | 0 | Neg | Neg | Pellagra encephalopathy,  cerebellar degeneration (Alc) |
| 35 | 0 | 1 | 0 | Not | 0 | 0 | 0 | Neg | Neg | Wernicke’s encephalopathy (MN) |
| 36 | 3 | 2 | 3 | Int | 3 | 0 | 0 | Neg | Neg | Cerebellar degeneration (Alc) |
| 37 | 0 | 1 | 0 | Not | 0 | 0 | 0 | Pos | Neg |  |
| 38 | 0 | 1 | 0 | Not | 1 | 0 | 0 | Neg | Pos |  |
| 39 | 1 | 1 | 2 | Low | 0 | 0 | 0 | Neg | Neg | Old cerebral infarction |
| 40 | 1 | 2 | 1 | Low | 2 | 0 | 0 | Neg | Neg |  |
| 41 | 3 | 3 | 3 | High | 0 | 1 | 1 | Neg | Neg |  |
| 42 | 1 | 1 | 1 | Low | 1 | 0 | 0 | Neg | Pos |  |
| 43 | 3 | 2 | 3 | Int | 1 | 1 | 0 | Neg | Neg |  |
| 44 | 2 | 3 | 2 | Int | 1 | 1 | 0 | Neg | Neg |  |
| 45 | 1 | 2 | 1 | Low | 0 | 1 | 0 | Neg | Pos | Old cerebral infarction |
| 46 | 1 | 2 | 1 | Low | 0 | 0 | 0 | Neg | Pos |  |
| 47 | 3 | 3 | 3 | High | 0 | 6 | 1 | Neg | Neg |  |
| 48 | 0 | 1 | 0 | Not | 0 | 0 | 0 | Neg | Neg |  |
| 49 | 1 | 1 | 1 | Low | 0 | 0 | 0 | Neg | Neg | Old brain contusion |
| 50 | 0 | 0 | 0 | Not | 0 | 0 | 0 | Neg | Neg |  |
| 51 | 0 | 0 | 0 | Not | 0 | 0 | 0 | Neg | Neg |  |
| 52 | 1 | 2 | 1 | Low | 0 | 4 | 0 | Neg | Neg |  |
| 53 | 1 | 3 | 1 | Low | 2 | 0 | 0 | Neg | Neg |  |
| 54 | 1 | 2 | 1 | Low | 3 | 0 | 0 | Pos | Neg |  |
| 55 | 3 | 3 | 2 | High | 0 | 0 | 0 | Neg | Neg |  |
| 56 | 1 | 1 | 2 | Low | 0 | 0 | 0 | Neg | Pos | Old cerebral infarction |
| 57 | 3 | 3 | 3 | High | 0 | 0 | 1 | Neg | Neg |  |
| 58 | 0 | 1 | 0 | Not | 0 | 0 | 0 | Neg | Neg | Old cerebral hemorrhage |
| 59 | 3 | 3 | 3 | High | 1 | 1 | 1 | Neg | Neg |  |
| 60 | 0 | 2 | 0 | Not | 0 | 1 | 0 | Neg | Pos |  |
| 61 | 0 | 1 | 0 | Not | 0 | 0 | 0 | Neg | Neg |  |
| 62 | 0 | 1 | 0 | Not | 0 | 0 | 0 | Neg | Neg |  |
| 63 | 0 | 1 | 0 | Not | 0 | 1 | 0 | Neg | Neg |  |
| 64 | 3 | 3 | 3 | High | 3 | 2 | 1 | Pos | Neg |  |
| 65 | 3 | 3 | 3 | High | 2 | 0 | 0 | Neg | Neg |  |
| 66 | 0 | 2 | 0 | Not | 0 | 0 | 0 | Neg | Neg | Pellagra encephalopathy (Alc) |
| 67 | 0 | 1 | 0 | Not | 2 | 0 | 0 | Neg | Neg | Chronic CO toxicity |
| 68 | 0 | 2 | 0 | Not | 1 | 1 | 0 | Neg | Neg |  |
| 69 | 0 | 2 | 0 | Not | 0 | 0 | 0 | Neg | Neg |  |
| 70 | 1 | 3 | 1 | Low | 0 | 0 | 0 | Neg | Neg | Pick disease |
| 71 | 1 | 1 | 1 | Low | 0 | 0 | 0 | Neg | Neg |  |
| 72 | 1 | 0 | 1 | Low | 0 | 0 | 0 | Neg | Neg |  |
| 73 | 3 | 2 | 3 | Int | 0 | 5 | 0 | Neg | Neg |  |
| 74 | 0 | 1 | 0 | Not | 0 | 0 | 0 | Neg | Neg |  |
| 75 | 0 | 3 | 0 | Low | 3 | 1 | 0 | Neg | Neg | Corticobasal degeneration |
| 76 | 0 | 1 | 0 | Not | 3 | 0 | 0 | Neg | Neg |  |
| 77 | 1 | 2 | 2 | Int | 2 | 0 | 0 | Pos | Neg |  |
| 78 | 3 | 3 | 3 | High | 3 | 5 | 0 | Neg | Neg | Old cerebellar infarction |
| 79 | 1 | 1 | 1 | Low | 0 | 0 | 0 | Neg | Neg |  |
| 80 | 0 | 1 | 0 | Not | 0 | 0 | 0 | Neg | Neg | Fresh and old subdural hemorrhage^a^ |
| 81 | 3 | 3 | 3 | High | 3 | 1 | 2 | Neg | Neg |  |
| 82 | 1 | 2 | 1 | Low | 2 | 0 | 0 | Pos | Neg |  |
| 83 | 1 | 1 | 2 | Low | 0 | 0 | 0 | Neg | Neg |  |
| 84 | 3 | 3 | 3 | High | 3 | 0 | 0 | Neg | Neg |  |
| 85 | 0 | 2 | 0 | Not | 3 | 1 | 0 | Neg | Neg |  |
| 86 | 0 | 1 | 0 | Not | 0 | 0 | 0 | Neg | Neg | Mild brain contusion |
| 87 | 2 | 2 | 2 | Int | 3 | 1 | 0 | Neg | Neg |  |
| 88 | 0 | 0 | 0 | Not | 0 | 0 | 0 | Neg | Neg |  |
| 89 | 0 | 1 | 0 | Not | 0 | 0 | 0 | Neg | Neg |  |
| 90 | 2 | 2 | 3 | Int | 2 | 1 | 0 | Neg | Neg |  |
| 91 | 0 | 2 | 0 | Not | 0 | 0 | 0 | Neg | Neg |  |
| 92 | 3 | 3 | 2 | High | 3 | 0 | 2 | Neg | Neg |  |
| 93 | 0 | 0 | 0 | Not | 0 | 0 | 0 | Neg | Neg |  |
| 94 | 0 | 2 | 0 | Not | 3 | 0 | 0 | Neg | Neg |  |
| 95 | 0 | 3 | 0 | Not | 0 | 0 | 0 | Pos | Neg |  |
| 96 | 3 | 3 | 2 | High | 2 | 0 | 0 | Neg | Neg |  |
| 97 | 0 | 2 | 0 | Not | 0 | 0 | 0 | Neg | Neg |  |
| 98 | 0 | 2 | 0 | Not | 2 | 0 | 0 | Neg | Neg |  |
| 99 | 3 | 3 | 3 | High | 0 | 0 | 0 | Neg | Neg | Meningioma |
| 100 | 2 | 2 | 3 | Int | 0 | 1 | 0 | Neg | Neg |  |
| 101 | 0 | 1 | 0 | Not | 0 | 0 | 0 | Neg | Neg |  |
| 102 | 1 | 2 | 1 | Low | 0 | 0 | 0 | Neg | Pos |  |
| 103 | 3 | 3 | 2 | High | 3 | 0 | 0 | Neg | Neg |  |
| 104 | 2 | 2 | 2 | Int | 0 | 0 | 0 | Neg | Neg |  |
| 105 | 1 | 2 | 1 | Low | 2 | 0 | 0 | Pos | Neg |  |
| 106 | 0 | 1 | 0 | Not | 0 | 0 | 0 | Neg | Neg |  |
| 107 | 0 | 2 | 0 | Not | 0 | 4 | 0 | Neg | Neg |  |
| 108 | 3 | 3 | 3 | High | 0 | 0 | 0 | Neg | Neg |  |
| 109 | 2 | 3 | 3 | Int | 0 | 0 | 0 | Neg | Neg |  |
| 110 | 2 | 2 | 2 | Int | 2 | 0 | 0 | Neg | Neg |  |
| 111 | 0 | 2 | 0 | Not | 0 | 0 | 0 | Neg | Neg |  |
| 112 | 3 | 3 | 3 | High | 0 | 1 | 0 | Neg | Neg |  |
| 113 | 0 | 0 | 0 | Not | 0 | 0 | 0 | Neg | Neg |  |
| 114 | 2 | 2 | 2 | Int | 0 | 0 | 0 | Neg | Neg |  |
| 115 | 3 | 3 | 2 | High | 2 | 0 | 0 | Neg | Neg |  |
| 116 | 2 | 3 | 3 | Int | 0 | 6 | 0 | Neg | Neg |  |
| 117 | 0 | 2 | 0 | Not | 0 | 3 | 0 | Neg | Neg |  |
| 118 | 0 | 1 | 0 | Not | 3 | 0 | 2 | Neg | Neg |  |
| 119 | 0 | 1 | 0 | Not | 0 | 0 | 0 | Neg | Neg |  |
| 120 | 1 | 2 | 2 | Int | 0 | 0 | 0 | Neg | Neg | Old thalamic infarction |
| 121 | 2 | 3 | 3 | Int | 0 | 0 | 0 | Neg | Neg |  |
| 122 | 2 | 3 | 2 | Int | 0 | 4 | 0 | Neg | Neg |  |
| 123 | 2 | 2 | 2 | Int | 0 | 0 | 0 | Neg | Neg |  |
| 124 | 1 | 1 | 0 | Low | 0 | 0 | 0 | Neg | Neg |  |
| 125 | 3 | 3 | 3 | High | 0 | 0 | 1 | Neg | Neg | *PSEN1* mutation |
| 126 | 1 | 2 | 1 | Low | 0 | 0 | 0 | Neg | Neg | Primary melanosis of the DN |
| 127 | 2 | 3 | 3 | Int | 3 | 0 | 2 | Neg | Neg |  |
| 128 | 2 | 3 | 2 | Int | 0 | 0 | 0 | Neg | Neg |  |
| 129 | 1 | 2 | 1 | Low | 0 | 0 | 0 | Pos | Neg |  |
| 130 | 1 | 2 | 2 | Int | 0 | 1 | 0 | Neg | Neg |  |
| 131 | 0 | 0 | 0 | Not | 0 | 0 | 0 | Neg | Neg |  |
| 132 | 2 | 2 | 3 | Int | 0 | 0 | 0 | Neg | Neg |  |
| 133 | 0 | 2 | 0 | Not | 0 | 0 | 0 | Neg | Neg |  |
| 134 | 3 | 3 | 3 | High | 3 | 1 | 0 | Neg | Neg |  |
| 135 | 1 | 1 | 0 | Low | 0 | 0 | 0 | Neg | Neg |  |
| 136 | 1 | 1 | 2 | Low | 0 | 4 | 0 | Neg | Neg | Old cerebral infarction |
| 137 | 3 | 2 | 3 | Int | 0 | 0 | 0 | Neg | Neg |  |
| 138 | 3 | 3 | 2 | High | 0 | 0 | 0 | Neg | Neg |  |
| 139 | 2 | 2 | 2 | Int | 3 | 0 | 0 | Neg | Neg |  |
| 140 | 0 | 1 | 0 | Not | 0 | 1 | 0 | Neg | Neg |  |
| 141 | 3 | 3 | 3 | High | 0 | 0 | 0 | Neg | Neg |  |
| 142 | 0 | 2 | 0 | Not | 0 | 0 | 0 | Neg | Neg |  |
| 143 | 2 | 2 | 3 | Int | 0 | 0 | 0 | Neg | Neg |  |
| 144 | 0 | 1 | 0 | Not | 0 | 0 | 0 | Neg | Neg |  |
| 145 | 1 | 2 | 2 | Int | 0 | 0 | 0 | Neg | Neg |  |
| 146 | 0 | 1 | 0 | Not | 0 | 0 | 0 | Neg | Neg |  |
| 147 | 1 | 2 | 1 | Low | 0 | 0 | 0 | Neg | Neg |  |
| 148 | 3 | 3 | 3 | High | 0 | 0 | 0 | Neg | Neg |  |
| 149 | 1 | 2 | 2 | Int | 0 | 0 | 0 | Neg | Neg |  |
| 150 | 0 | 2 | 0 | Not | 3 | 0 | 0 | Neg | Neg |  |
| 151 | 0 | 2 | 0 | Not | 3 | 0 | 0 | Neg | Neg |  |
| 152 | 0 | 1 | 0 | Not | 0 | 0 | 0 | Neg | Neg |  |
| 153 | 0 | 2 | 0 | Not | 1 | 0 | 0 | Neg | Pos |  |
| 154 | 0 | 2 | 0 | Not | 0 | 0 | 0 | Neg | Pos |  |
| 155 | 3 | 3 | 3 | High | 0 | 1 | 1 | Neg | Neg |  |
| 156 | 2 | 1 | 2 | Low | 0 | 0 | 0 | Neg | Pos |  |
| 157 | 3 | 2 | 2 | Int | 1 | 1 | 0 | Neg | Neg |  |
| 158 | 0 | 1 | 0 | Not | 0 | 0 | 0 | Neg | Neg |  |
| 159 | 3 | 2 | 3 | Int | 0 | 0 | 1 | Neg | Neg |  |
| 160 | 3 | 2 | 2 | Int | 1 | 0 | 0 | Neg | Neg |  |
| 161 | 1 | 1 | 0 | Low | 0 | 0 | 0 | Neg | Neg |  |
| 162 | 3 | 2 | 2 | Int | 3 | 0 | 1 | Neg | Neg |  |
| 163 | 2 | 2 | 2 | Int | 3 | 0 | 0 | Pos | Neg |  |
| 164 | 2 | 2 | 2 | Int | 2 | 3 | 0 | Neg | Neg |  |
| 165 | 1 | 2 | 1 | Low | 0 | 5 | 0 | Neg | Neg |  |
| 166 | 1 | 2 | 0 | Low | 2 | 0 | 0 | Neg | Neg |  |
| 167 | 1 | 1 | 1 | Low | 0 | 0 | 0 | Neg | Neg |  |
| 168 | 0 | 1 | 0 | Not | 0 | 4 | 0 | Neg | Neg |  |

**Abbreviations:** AGD, argyrophilic grain disease; Alc, (chronic) alcoholism-related; int, intermediate; DN, dentate nucleus; LATE-NC, limbic-predominant age-related TDP-43 encephalopathy; LBD, Lewy body disease; MN, malnutrition-related (non-alcoholism case); PSP, progressive supranuclear palsy

^a^ In this case, because there were no severe external injuries, it was not included in the cases of trauma caused by falls.

**Table S3.** Comparison of results between the indoor and outdoor hypothermia case groups.

|  | Indoor cases (N = 47) | Outdoor cases (N = 121) | *p* value^a^ |
| --- | --- | --- | --- |
| Age ± SD (range) | 72.3 ± 14.4 (32–93) | 74.6 ± 14.2 (24–99) | 0.28 |
| Sex (F/M) | 21/26 | 54/67 | 1 |
| Cognitive impairment-positive (%) | 12 (30)^b^ | 48 (42)^c^ | **0.0498** |
| AD pathology-positive (%) | 15 (32) | 52 (43) | 0.22 |
| AGD pathology-positive (%) | 10 (21) | 42 (35) | 0.10 |
| LBD pathology-positive (%) | 13 (28) | 26 (21) | 0.42 |
| LATE-NC pathology-positive (%) | 3 (6) | 12 (10) | 0.56 |

**Boldface** signifies the values that are significant at *p* < 0.05.

^a^ Hypothermia vs. other causes, evaluated using Fisher’s exact test or Mann–Whitney U-test.

^b^ Frequency among the 40 cases for which clinical information was available.

^c^ Frequency among the 113 cases for which clinical information was available.

**Table S4.** Summary of the neuropathological findings in patients with CI.

|  | All  (N = 60) | Female  (N = 31) | Male  (N = 29) | *p* value^a^ |
| --- | --- | --- | --- | --- |
| Mean age (range) | 83.1 ± 7.2 (57–99) | 84.7 ± 6.2 (72–99) | 81.3 ± 7.7 (57–92) | 0.17 |
| 1 pathology (%) | 20 (33) | 11 (35) | 9 (31) | 0.79 |
| AD | 14 (23) | 7 (23) | 7 (24) | 1 |
| AGD | 2 (3) | 2 (6) | 0 | 0.49 |
| Vascular | 2 (3) | 1 (3) | 1 (3) | 1 |
| CBD | 1 (2) | 0 | 1 (3) | 0.48 |
| PSP | 1 (2) | 1 (3) | 0 | 1 |
| 2 pathologies (%) | 24 (40) | 12 (39) | 12 (41) | 0.79 |
| AD/AGD | 8 (13) | 4 (13) | 4 (14) | 1 |
| AD/LBD | 4 (7) | 3 (10) | 1 (3) | 0.61 |
| AD/LATE-NC | 3 (5) | 2 (6) | 1 (3) | 1 |
| AD/PSP | 2 (3) | 0 | 2 (7) | 0.23 |
| AGD/Vascular | 3 (5) | 2 (6) | 1 (3) | 1 |
| AGD/LATE-NC | 1 (2) | 0 | 1 (3) | 0.48 |
| AGD/Other^b^ | 1 (2) | 0 | 1 (3) | 0.48 |
| LBD/Vascular | 2 (3) | 1 (3) | 1 (3) | 1 |
| 3 pathologies (%) | 13 (22) | 8 (26) | 5 (17) | 0.54 |
| AD/AGD/LBD | 6 (10) | 4 (13) | 2 (7) | 0.67 |
| AD/LBD/LATE-NC | 3 (5) | 1 (3) | 2 (7) | 0.61 |
| AD/AGD/LATE-NC | 2 (3) | 1 (3) | 1 (3) | 1 |
| AD/AGD/PSP | 1 (2) | 1 (3) | 0 | 1 |
| AGD/CBD/LBD | 1 (2) | 1 (3) | 0 | 1 |
| 4 pathologies (%) |  |  |  |  |
| AD/AGD/LATE-NC/LBD | 2 (2) | 0 | 2 (7) | 0.23 |
| 5 pathologies (%) |  |  |  |  |
| AD/AGD/LATE-NC/LBD/PSP | 1 (2) | 0 | 1 (3) | 0.48 |

^a^ Females vs. males, evaluated using Fisher’s exact test.

^b^ AGD and chronic CO toxicity.

**Table S5.** All neuropathological findings in cases with CI.

|  | Age | Sex | A | B | C | AD level | AGD | LBD | LATE-NC | PSP | Vascular | Cause(s) of CI |
| --- | --- | --- | --- | --- | --- | --- | --- | --- | --- | --- | --- | --- |
| 1 | 88 | M | 3 | 3 | 3 | high | 0 | 5 | 0 | Neg | Neg | AD/LBD |
| 2 | 82 | M | 1 | 2 | 2 | int | 0 | 0 | 0 | Neg | Neg | AD |
| 3 | 78 | M | 1 | 3 | 2 | int | 0 | 0 | 0 | Pos | Neg | AD/PSP |
| 4 | 85 | M | 3 | 3 | 3 | high | 0 | 0 | 0 | Pos | Neg | AD/PSP |
| 5 | 84 | F | 3 | 3 | 3 | high | 0 | 0 | 0 | Neg | Neg | AD |
| 6 | 65 | M | 0 | 3 | 0 | not | 0 | 0 | 0 | Neg | Neg | CBD |
| 7 | 78 | F | 3 | 3 | 3 | high | 0 | 0 | 1 | Neg | Neg | AD/LATE-NC |
| 8 | 84 | M | 3 | 2 | 3 | int | 3 | 0 | 0 | Neg | Neg | AD/AGD |
| 9 | 78 | F | 0 | 1 | 0 | not | 1 | 0 | 0 | Neg | Pos | Vascular/AGD |
| 10 | 99 | F | 1 | 2 | 1 | low | 2 | 0 | 0 | Neg | Neg | AGD |
| 11 | 84 | F | 3 | 3 | 3 | high | 0 | 1 | 1 | Neg | Neg | AD/LBD/LATE-NC |
| 12 | 86 | F | 1 | 1 | 1 | low | 1 | 0 | 0 | Neg | Pos | Vascular/AGD |
| 13 | 91 | F | 3 | 2 | 3 | int | 1 | 1 | 0 | Neg | Neg | AD/LBD/AGD |
| 14 | 85 | M | 1 | 2 | 1 | low | 0 | 1 | 0 | Neg | Pos | LBD/Vascular |
| 15 | 80 | M | 3 | 3 | 3 | high | 0 | 6 | 1 | Neg | Neg | AD/LBD/LATE-NC |
| 16 | 91 | F | 3 | 3 | 2 | high | 0 | 0 | 0 | Neg | Neg | AD |
| 17 | 86 | M | 3 | 3 | 3 | high | 0 | 0 | 1 | Neg | Neg | AD/LATE-NC |
| 18 | 75 | M | 3 | 3 | 3 | high | 1 | 1 | 1 | Neg | Neg | AD/LBD/LATE-NC/AGD |
| 19 | 75 | F | 0 | 2 | 0 | not | 0 | 1 | 0 | Neg | Pos | Vascular/LBD |
| 20 | 89 | M | 3 | 3 | 3 | high | 3 | 2 | 1 | Pos | Neg | AD/PSP/AGD/LBD/LATE-NC |
| 21 | 85 | M | 3 | 3 | 3 | high | 2 | 0 | 0 | Neg | Neg | AD/AGD |
| 22 | 66 | M | 0 | 1 | 0 | not | 2 | 0 | 0 | Neg | Neg | chronic CO toxicity/AGD |
| 23 | 93 | F | 3 | 2 | 3 | int | 0 | 5 | 0 | Neg | Neg | AD/LBD |
| 24 | 80 | F | 0 | 3 | 0 | low | 3 | 1 | 0 | Neg | Neg | CBD/AGD/LBD |
| 25 | 86 | F | 3 | 3 | 3 | high | 3 | 5 | 0 | Neg | Neg | AD/LBD/AGD |
| 26 | 83 | M | 3 | 3 | 3 | high | 3 | 1 | 2 | Neg | Neg | AD/AGD/LBD/LATE-NC |
| 27 | 81 | M | 3 | 3 | 3 | high | 3 | 0 | 0 | Neg | Neg | AD/AGD |
| 28 | 83 | M | 2 | 2 | 2 | int | 3 | 1 | 0 | Neg | Neg | AD/AGD/LBD |
| 29 | 76 | M | 2 | 2 | 3 | int | 2 | 1 | 0 | Neg | Neg | AD/AGD/LBD |
| 30 | 82 | F | 3 | 3 | 2 | high | 3 | 0 | 2 | Neg | Neg | AD/AGD/LATE-NC |
| 31 | 91 | F | 3 | 3 | 2 | high | 2 | 0 | 0 | Neg | Neg | AD/AGD |
| 32 | 87 | M | 3 | 3 | 3 | high | 0 | 0 | 0 | Neg | Neg | AD |
| 33 | 84 | F | 3 | 3 | 2 | high | 3 | 0 | 0 | Neg | Neg | AD/AGD |
| 34 | 93 | F | 2 | 2 | 2 | int | 2 | 0 | 0 | Neg | Neg | AD/AGD |
| 35 | 85 | F | 3 | 3 | 2 | high | 2 | 0 | 0 | Neg | Neg | AD/AGD |
| 36 | 76 | F | 2 | 3 | 3 | int | 0 | 6 | 0 | Neg | Neg | AD/LBD |
| 37 | 86 | M | 0 | 1 | 0 | not | 3 | 0 | 2 | Neg | Neg | AGD/LATE-NC |
| 38 | 72 | F | 1 | 2 | 2 | int | 0 | 0 | 0 | Neg | Neg | AD |
| 39 | 75 | M | 2 | 3 | 3 | int | 0 | 0 | 0 | Neg | Neg | AD |
| 40 | 86 | F | 2 | 3 | 2 | int | 0 | 4 | 0 | Neg | Neg | AD/LBD |
| 41 | 82 | F | 2 | 2 | 2 | int | 0 | 0 | 0 | Neg | Neg | AD |
| 42 | 57 | M | 3 | 3 | 3 | high | 0 | 0 | 1 | Neg | Neg | AD/LATE-NC |
| 43 | 85 | F | 2 | 3 | 2 | int | 0 | 0 | 0 | Neg | Neg | AD |
| 44 | 88 | F | 1 | 2 | 1 | low | 0 | 0 | 0 | Pos | Neg | PSP |
| 45 | 86 | M | 3 | 3 | 2 | high | 0 | 0 | 0 | Neg | Neg | AD |
| 46 | 79 | F | 2 | 2 | 2 | int | 3 | 0 | 0 | Neg | Neg | AD/AGD |
| 47 | 92 | M | 3 | 3 | 3 | high | 0 | 0 | 0 | Neg | Neg | AD |
| 48 | 86 | M | 1 | 2 | 2 | int | 0 | 0 | 0 | Neg | Neg | AD |
| 49 | 90 | F | 3 | 3 | 3 | high | 0 | 0 | 0 | Neg | Neg | AD |
| 50 | 75 | F | 1 | 2 | 2 | int | 0 | 0 | 0 | Neg | Neg | AD |
| 51 | 78 | M | 0 | 2 | 0 | not | 3 | 0 | 0 | Neg | Neg | AGD |
| 52 | 84 | M | 0 | 2 | 0 | not | 1 | 0 | 0 | Neg | Pos | Vascular/AGD |
| 53 | 87 | M | 0 | 2 | 0 | not | 0 | 0 | 0 | Neg | Pos | Vascular |
| 54 | 86 | F | 3 | 3 | 3 | high | 0 | 1 | 1 | Neg | Neg | AD/LBD/LATE-NC |
| 55 | 92 | F | 2 | 1 | 2 | low | 0 | 0 | 0 | Neg | Pos | Vascular |
| 56 | 81 | F | 3 | 2 | 2 | int | 1 | 1 | 0 | Neg | Neg | AD/LBD/AGD |
| 57 | 79 | M | 3 | 2 | 3 | int | 0 | 0 | 1 | Neg | Neg | AD/LATE-NC |
| 58 | 90 | F | 3 | 2 | 2 | int | 3 | 0 | 1 | Neg | Neg | AD/AGD/LATE-NC |
| 59 | 84 | F | 2 | 2 | 2 | int | 3 | 0 | 0 | Pos | Neg | AD/PSP/AGD |
| 60 | 88 | M | 2 | 2 | 2 | int | 2 | 3 | 0 | Neg | Neg | AD/LBD/AGD |

**Table S6.** All neuropathological findings in cases presumed to have developed lethal hypothermia while wandering.

|  | Age | Sex | S/D^a^ | A | B | C | AD level | AGD | LBD | LATE-NC | PSP | Vascular | Cause(s) of CI |
| --- | --- | --- | --- | --- | --- | --- | --- | --- | --- | --- | --- | --- | --- |
| 1 | 88 | M | OD | 3 | 3 | 3 | high | 0 | 5 | 0 | Neg | Neg | AD/LBD |
| 2 | 82 | M | OD | 1 | 2 | 2 | int | 0 | 0 | 0 | Neg | Neg | AD |
| 3 | 65 | M | OD | 3 | 3 | 3 | high | 0 | 1 | 1 | Neg | Neg | CBD |
| 4 | 84 | F | ID | 3 | 3 | 2 | high | 0 | 0 | 0 | Neg | Neg | AD/LBD/LATE-NC |
| 5 | 91 | F | OD | 3 | 3 | 3 | high | 1 | 1 | 1 | Neg | Neg | AD |
| 6 | 75 | M | OD | 3 | 3 | 3 | high | 3 | 2 | 1 | Positive | Neg | AD/LBD/LATE-NC/AGD |
| 7 | 89 | M | OD | 3 | 3 | 3 | high | 2 | 0 | 0 | Neg | Neg | AD/PSP/AGD/LBD/LATE-NC |
| 8 | 85 | M | OD | 3 | 2 | 3 | int | 0 | 5 | 0 | Neg | Neg | AD/AGD |
| 9 | 83 | M | OD | 3 | 3 | 3 | high | 3 | 0 | 0 | Neg | Neg | AD/AGD/LBD/LATE-NC |
| 10 | 81 | M | OD | 2 | 2 | 3 | int | 2 | 1 | 0 | Neg | Neg | AD/AGD |
| 11 | 76 | M | OD | 3 | 3 | 2 | high | 3 | 0 | 2 | Neg | Neg | AD/AGD/LBD |
| 12 | 82 | F | OD | 3 | 3 | 3 | high | 0 | 0 | 0 | Neg | Neg | AD/AGD/LATE-NC |
| 13 | 87 | M | OD | 2 | 2 | 2 | int | 2 | 0 | 0 | Neg | Neg | AD |
| 14 | 93 | F | OD | 3 | 3 | 2 | high | 2 | 0 | 0 | Neg | Neg | AD/AGD |
| 15 | 85 | F | OD | 2 | 3 | 3 | int | 0 | 6 | 0 | Neg | Neg | AD/AGD |
| 16 | 76 | F | OD | 2 | 3 | 3 | int | 0 | 0 | 0 | Neg | Neg | AD/LBD |
| 17 | 75 | M | ID | 2 | 3 | 2 | int | 0 | 4 | 0 | Neg | Neg | AD |
| 18 | 86 | F | ID | 3 | 3 | 3 | high | 0 | 0 | 1 | Neg | Neg | AD/LBD |
| 19 | 57 | M | ID | 2 | 3 | 2 | int | 0 | 0 | 0 | Neg | Neg | AD/LATE-NC |
| 20 | 89 | M | OD | 3 | 3 | 3 | high | 0 | 0 | 0 | Neg | Neg | AD |
| 21 | 88 | F | OD | 1 | 2 | 2 | int | 0 | 0 | 0 | Neg | Neg | AD |
| 22 | 79 | F | OD | 1 | 2 | 2 | int | 0 | 0 | 0 | Neg | Neg | AD |
| 23 | 92 | M | OD | 3 | 3 | 3 | high | 0 | 1 | 1 | Neg | Neg | AD |
| 24 | 90 | F | OD | 3 | 2 | 2 | int | 1 | 1 | 0 | Neg | Neg | AD |
| 25 | 75 | F | OD | 3 | 2 | 3 | int | 0 | 0 | 1 | Neg | Neg | AGD |
| 26 | 84 | M | OD | 3 | 2 | 2 | int | 3 | 0 | 1 | Neg | Neg | Vascular dementia |
| 27 | 87 | M | OD | 3 | 3 | 3 | high | 0 | 5 | 0 | Neg | Neg | AD/LBD/LATE-NC |
| 28 | 86 | F | OD | 1 | 2 | 2 | int | 0 | 0 | 0 | Neg | Neg | Vascular dementia |
| 29 | 92 | F | OD | 3 | 3 | 3 | high | 0 | 1 | 1 | Neg | Neg | AD/LBD/AGD |
| 30 | 81 | F | OD | 3 | 3 | 2 | high | 0 | 0 | 0 | Neg | Neg | AD/LATE-NC |
| 31 | 79 | M | OD | 3 | 3 | 3 | high | 1 | 1 | 1 | Neg | Neg | AD/AGD/LATE-NC |

^a^ All indoor cases were discovered in buildings other than the patient's home.

**Table S7.** All neuropathological findings in cases with several traumatic injuries associated with falls.

|  | Age | Sex | M/D | S/D | F/H injury^a^ | BF | CI | B-EtOH | Drug | A | B | C | AD level | AGD | LBD | LATE-NC | PSP | Vascular | Cause(s) of CI |
| --- | --- | --- | --- | --- | --- | --- | --- | --- | --- | --- | --- | --- | --- | --- | --- | --- | --- | --- | --- |
| 1 | 78 | M | Acc | OD | Present | Pos | Pos | 0 | Neg | 1 | 3 | 2 | int | 0 | 0 | 0 | Pos | Neg | AD/PSP |
| 2 | 85 | M | Ill | ID | Present | Pos | Pos | 0 | Neg | 3 | 3 | 3 | high | 0 | 0 | 0 | Pos | Neg | AD/PSP |
| 3 | 61 | F | Acc | OD | Present | Pos | Neg | 0 | BZO | 0 | 1 | 0 | not | 0 | 0 | 0 | Neg | Neg | NA |
| 4 | 83 | M | Acc | OD | Present | Neg | NA | 0 | Neg | 2 | 2 | 3 | int | 0 | 0 | 0 | Neg | Neg | NA |
| 5 | 84 | F | Acc | OD | Present | Pos | Pos | 0 | Neg | 3 | 3 | 3 | high | 0 | 0 | 0 | Neg | Neg | AD |
| 6^b^ | 64 | M | Ill | ID | Present | Pos | Neg | 0 | TCA | 0 | 1 | 0 | not | 0 | 0 | 0 | Neg | Neg | NA |
| 7 | 82 | F | Ill | ID | Present | Neg | Neg | 0 | Neg | 1 | 1 | 1 | low | 1 | 0 | 0 | Neg | Neg | NA |
| 8 | 91 | F | Sui | OD | Present | Pos | Pos | 0 | Neg | 3 | 2 | 3 | int | 1 | 1 | 0 | Neg | Neg | AD/LBD/AGD |
| 9 | 85 | M | Acc | OD | Absent | Pos | NA | 0 | Neg | 2 | 3 | 2 | int | 1 | 1 | 0 | Neg | Neg | NA |
| 10 | 78 | F | Acc | OD | Absent | Pos | Neg | 0 | Neg | 1 | 2 | 1 | low | 0 | 0 | 0 | Neg | Pos | NA |
| 11 | 89 | F | Acc | OD | Present | Neg | Neg | 0 | Neg | 1 | 3 | 1 | low | 2 | 0 | 0 | Neg | Neg | NA |
| 12 | 76 | F | Acc | OD | Present | Neg | Neg | 0 | Neg | 1 | 1 | 2 | low | 0 | 0 | 0 | Neg | Pos | NA |
| 13 | 86 | M | Acc | OD | Present | Pos | Pos | 0 | Neg | 3 | 3 | 3 | high | 0 | 0 | 1 | Neg | Neg | AD/LATE-NC |
| 14 | 66 | M | Acc | OD | Present | Pos | Neg | 0 | Neg | 0 | 1 | 0 | not | 2 | 0 | 0 | Neg | Neg | Chronic CO toxicity/AGD |
| 15 | 65 | M | Acc | OD | Present | Pos | Neg | 2.194 | Neg | 1 | 1 | 1 | low | 0 | 0 | 0 | Neg | Neg | NA |
| 16 | 80 | F | Sui | OD | Present | Pos | Pos | 0 | OPI/BZO | 0 | 3 | 0 | low | 3 | 1 | 0 | Neg | Neg | CBD/AGD/LBD |
| 17^c^ | 73 | F | Acc | ID | Present | Neg | Neg | 0 | Neg | 0 | 1 | 0 | not | 0 | 0 | 0 | Neg | Neg | NA |
| 18 | 83 | M | Acc | ID | Present | Pos | Pos | 0 | Neg | 2 | 2 | 2 | int | 3 | 1 | 0 | Neg | Neg | AD/AGD/LBD |
| 19 | 71 | F | Sui | OD | Absent | Pos | Neg | 0 | BZO | 0 | 2 | 0 | not | 0 | 0 | 0 | Neg | Neg | NA |
| 20 | 87 | M | Acc | OD | Present | Neg | Pos | 0 | Neg | 3 | 3 | 3 | high | 0 | 0 | 0 | Neg | Neg | AD |
| 21 | 67 | M | Acc | OD | Present | Pos | Neg | 1 | Neg | 2 | 2 | 3 | int | 0 | 1 | 0 | Neg | Neg | NA |
| 22 | 84 | F | Unk | OD | Absent | Pos | Pos | 0 | Neg | 3 | 3 | 2 | high | 3 | 0 | 0 | Neg | Neg | AD/AGD^c^ |
| 23 | 59 | M | Acc | OD | Present | Pos | Neg | 0 | Neg | 2 | 2 | 2 | int | 0 | 0 | 0 | Neg | Neg | NA |
| 24 | 69 | F | Acc | OD | Present | Pos | Neg | 0 | Neg | 1 | 2 | 1 | low | 2 | 0 | 0 | Pos | Neg | NA |
| 25 | 80 | F | Acc | ID | Absent | Pos | Neg | 0 | Neg | 3 | 3 | 3 | high | 0 | 0 | 0 | Neg | Neg | NA |
| 26 | 91 | F | Acc | ID | Absent | Pos | NA | 0 | Neg | 2 | 3 | 3 | int | 0 | 0 | 0 | Neg | Neg | NA |
| 27 | 75 | M | Acc | OD | Absent | Neg | Neg | 0 | Neg | 0 | 2 | 0 | not | 0 | 0 | 0 | Neg | Neg | NA |
| 28 | 76 | F | Acc | OD | Present | Pos | Pos | 0 | Neg | 2 | 3 | 3 | int | 0 | 6 | 0 | Neg | Neg | AD/LBD |
| 29 | 75 | M | Acc | ID | Absent | Pos | Pos | 0 | Neg | 2 | 3 | 3 | int | 0 | 0 | 0 | Neg | Neg | AD |
| 30 | 77 | F | Acc | OD | Present | Pos | Neg | 0 | Neg | 2 | 3 | 3 | int | 3 | 0 | 2 | Neg | Neg | NA |
| 31 | 79 | F | Sui | OD | Absent | Pos | Neg | 0 | BZO | 1 | 2 | 2 | int | 0 | 1 | 0 | Neg | Neg | NA |
| 32 | 88 | F | Acc | OD | Present | Pos | Pos | 0 | Neg | 3 | 3 | 2 | high | 0 | 0 | 0 | Neg | Neg | AD |
| 33 | 72 | F | Sui | OD | Absent | Pos | Neg | 0 | Neg | 1 | 2 | 1 | low | 0 | 0 | 0 | Neg | Neg | NA |
| 34 | 72 | M | Acc | ID | Present | Pos | Neg | 0 | Neg | 0 | 2 | 0 | not | 3 | 0 | 0 | Neg | Neg | NA |
| 35 | 85 | M | Acc | ID | Absent | Pos | Neg | 0 | Neg | 1 | 2 | 0 | low | 2 | 0 | 0 | Neg | Neg | AD/PSP |

**Abbreviation**: BF, Bone fracture; F/H, face and/or head

^a^ Presence or absence of facial and/or head injuries—indicators of external force that may have caused concussion or loss of consciousness.

^b^ This case had classic-type superficial hemosiderosis, indicating ataxia as the presumed cause of their fall.

^c^ This case exhibited a small hemorrhagic lesion in a right caudate nucleus and a mild cerebral contusion, with the former likely causing a fall, which resulted in immobility and subsequent hypothermia.


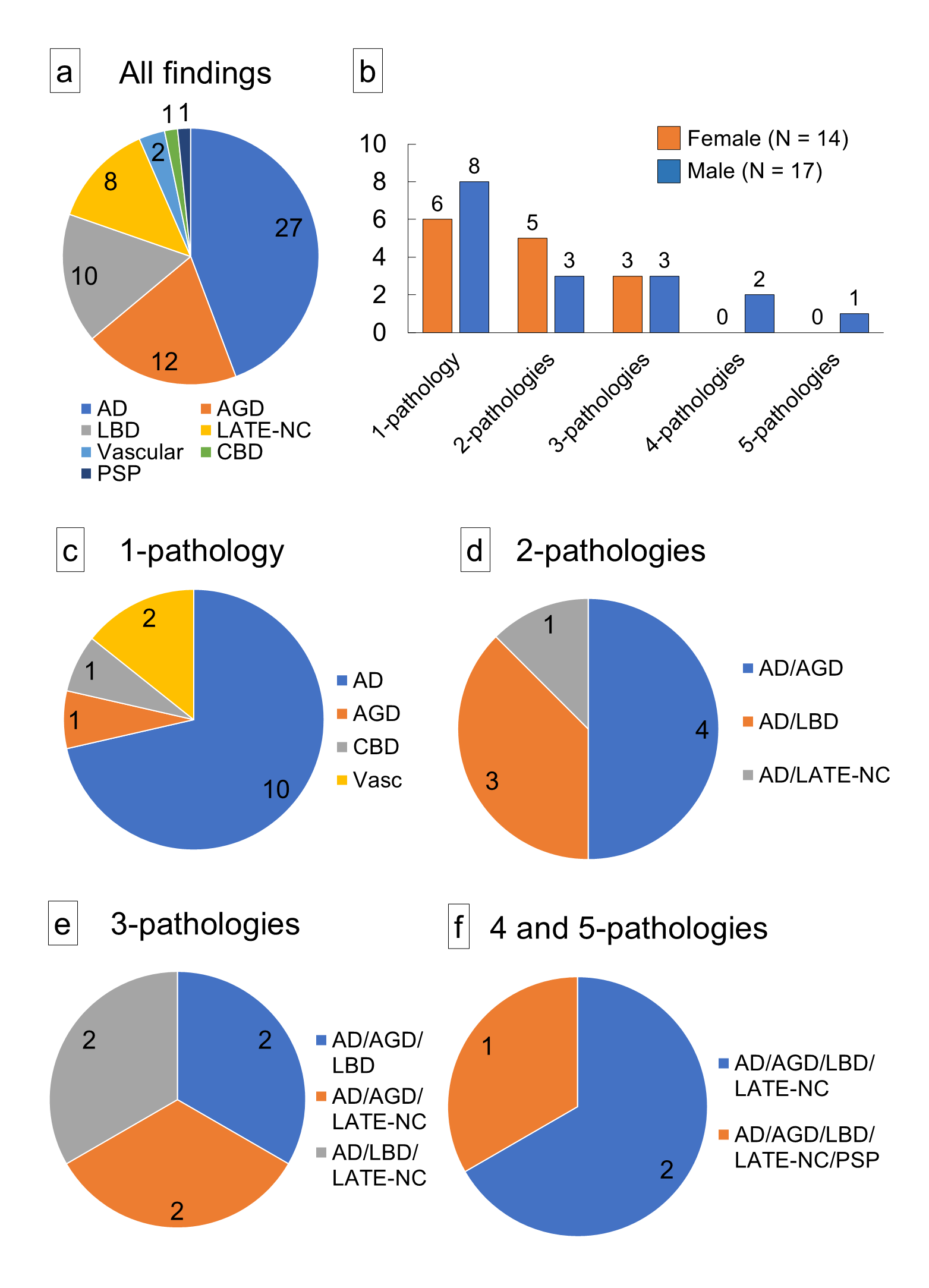
**Figure S1.** Neuropathological findings in patients with cognitive impairment (CI), who presumably developed hypothermia while wandering.

(a) All neuropathological findings. (b) Frequency of combinations of neuropathological diseases. Frequency of CI-causative diseases in groups with one (c), two (d), three (e), and four and five pathologies (f).


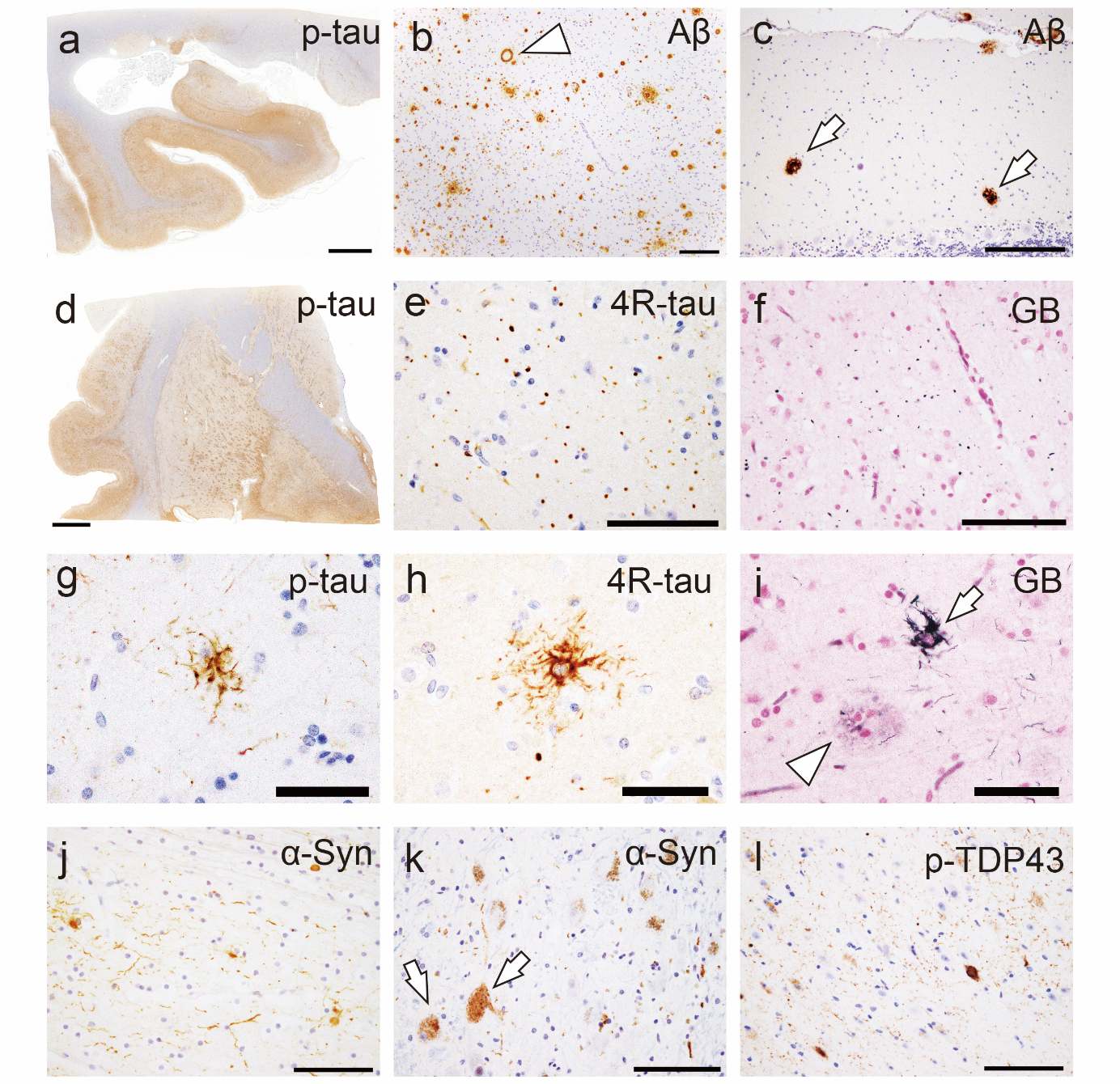
**Figure S2.** Representative neuropathological findings in the CI-positive case with five pathologies.

(a) Hippocampal region; (b) frontal cortex; (c) cerebellum; (d) basal ganglia and insular cortex; (e, f) insular cortex; (g–i) motor cortex; (j) olfactory bulb; (k) substantia nigra; (l) subiculum. Immunohistochemistry for phosphorylated (p-) tau (AT8) (a, d, g); amyloid-beta (Aβ) (b, c); four-repeat (4R-) tau (e, h); α-synuclein (α-Syn) (j, k); p-TDP43 (l). Gallyas–Braak (GB) staining (f, j).

(a) p-tau-positive deposits are diffusely observed from the hippocampus to the occipito-temporal gyrus. Additional examination confirmed p-tau-positive deposits in the striate area (Braak’s neurofibrillary score VI).^17^ Numerous senile plaques are present in the neocortex (CERAD score frequent),^18^ and Aβ deposition is observed in the cerebellum (arrow) (Thal’s Aβ stage 5).^19^ (d) Severe p-tau deposition is observed in the basal ganglia and insular cortex. (e, f) Numerous grains are observed (Saito’s AGD stage III).^24^ (g–i) Tufted astrocytes are observed (arrow indicates tufted astrocyte, arrowhead indicates senile plaque). (j, k) Lewy-related pathology is observed up to the midbrain (Braak’s Lewy-related pathology stage 3).^22^ (l) p-TDP43 pathology is observed up to the hippocampal region (LATE-NC pathology stage 2).^27,28^

Scale bar = 3 mm (a, d); 200 μm (b, c); 100 μm (e, f, j–l); 50 μm (g–i
